# Correction: Differences in Muscle Transcriptome among Pigs Phenotypically Extreme for Fatty Acid Composition

**DOI:** 10.1371/journal.pone.0103668

**Published:** 2014-07-21

**Authors:** The PLOS ONE Staff

## Notice of Republication

This article was republished on June 27, 2014, to correct an error in the byline. The publisher apologizes for the error. Please download this article again to view the correct version. The originally published, uncorrected article and the republished, corrected article are provided here for reference.

## Supporting Information

File S1 Originally published, uncorrected article.
(PDF)

File S2 Republished, corrected article.
(PDF)
