# Supplementary figures and images for: Differences in Muscle Transcriptome among Pigs Phenotypically Extreme for Fatty Acid Composition
Source: PLoS One. 2014 Jun 13;9(6):e99720. doi: 10.1371/journal.pone.0099720 (PMC4057286; doi:10.1371/journal.pone.0099720)

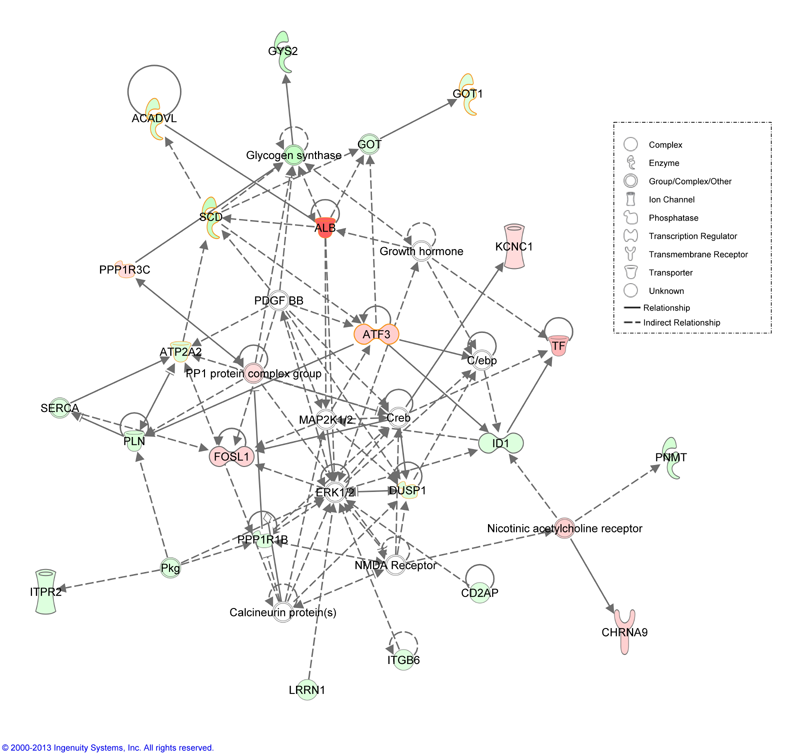

Supplement: Figure S1 — Network (indirect, score 36) generated by IPA of 35 focus genes corresponding to metabolic disease, lipid metabolism and molecular transport. Node colours indicate gene expression, being the red nodes higher-expressed genes and the green nodes lower-expressed genes in the H group relative to the L group. Colour intensity is related to the degree of expression. Node shapes indicate the biological function of the protein. (TIF) [file pone.0099720.s001.tif]
