# Supplementary material for: Differences in Muscle Transcriptome among Pigs Phenotypically Extreme for Fatty Acid Composition
Source: PLoS One. 2014 Jun 13;9(6):e99720. doi: 10.1371/journal.pone.0099720 (PMC4057286; doi:10.1371/journal.pone.0099720)
Supplement: Table S1 — Percentage of reads mapped for each sample and their localization (exonic, intronic or intergenic) regarding the pig reference genome sequence. (DOCX) [file pone.0099720.s002.docx]

**Table S1.** Percentage of reads mapped for each sample and their localization (exonic, intronic or intergenic) regarding the pig reference genome sequence (Sscrofa10.2 genome assembly).

| Sample | Mapped Reads (%) | Exonic Reads (%) | Intronic Reads (%) | Intergenic Reads (%) |
| --- | --- | --- | --- | --- |
| H1 | 84.70 | 78.74 | 6.14 | 15.12 |
| H2 | 85.21 | 80.87 | 5.29 | 13.84 |
| H3 | 85.80 | 79.69 | 5.94 | 14.37 |
| H4 | 85.09 | 77.51 | 6.77 | 15.71 |
| H5 | 86.11 | 84.01 | 3.61 | 12.37 |
| H6 | 86.65 | 80.03 | 5.67 | 14.31 |
| L1 | 76.48 | 78.16 | 6.58 | 15.26 |
| L2 | 86.64 | 79.22 | 5.80 | 14.98 |
| L3 | 85.53 | 78.14 | 6.47 | 15.38 |
| L4 | 85.14 | 77.70 | 6.84 | 15.45 |
| L5 | 85.63 | 77.87 | 6.17 | 15.97 |
| L6 | 85.49 | 77.91 | 6.19 | 15.90 |
| Average | 84.87 | 79.15 | 5.96 | 14.89 |
