# Supplementary material for: Differences in Muscle Transcriptome among Pigs Phenotypically Extreme for Fatty Acid Composition
Source: PLoS One. 2014 Jun 13;9(6):e99720. doi: 10.1371/journal.pone.0099720 (PMC4057286; doi:10.1371/journal.pone.0099720)
Supplement: Table S2 — Total number of assembled transcripts with cufflinks. (DOCX) [file pone.0099720.s003.docx]

| **Table S2.** Total number of assemble transcripts with cufflinks. | | | | | | | | | | | | | | | | | | | | | | | | | | |  |  | |
| --- | --- | --- | --- | --- | --- | --- | --- | --- | --- | --- | --- | --- | --- | --- | --- | --- | --- | --- | --- | --- | --- | --- | --- | --- | --- | --- | --- | --- | --- |
|  | | | | | | | | | | | | | | | | | | | | | | | | | | |  |  |  |
| **Animal** | **L1** | | | **L2** | | **L3** | | **L4** | | **L5** | | **L6** | | **H1** | | **H2** | | **H3** | | **H4** | | **H5** | | **H6** | | **Total** | **Mean** | |  |
|  | ***TA*** | | ***%*** | ***TA*** | ***%*** | ***TA*** | ***%*** | ***TA*** | ***%*** | ***TA*** | ***%*** | ***TA*** | ***%*** | ***TA*** | ***%*** | ***TA*** | ***%*** | ***TA*** | ***%*** | ***TA*** | ***%*** | ***TA*** | ***%*** | ***TA*** | ***%*** |  |  |  |  |
| = | 25,990 | | 61 | 25,989 | 60 | 26,136 | 66 | 25,998 | 60 | 25,957 | 59 | 25,958 | 56 | 25,955 | 57 | 25,875 | 53 | 26,029 | 69 | 25,976 | 61 | 25,994 | 59 | 26,028 | 65 | 311,885 | 25,990 | |  |
| c | 3 | | 0 | 6 | 0 | 7 | 0 | 5 | 0 | 6 | 0 | 5 | 0 | 5 | 0 | 2 | 0 | 4 | 0 | 3 | 0 | 2 | 0 | 4 | 0 | 52 | 4 | |  |
| e | 724 | | 2 | 741 | 2 | 582 | 1 | 778 | 2 | 778 | 2 | 945 | 2 | 911 | 2 | 938 | 2 | 258 | 1 | 722 | 2 | 792 | 2 | 522 | 1 | 8,691 | 724 | |  |
| i | 1,452 | | 3 | 1,594 | 4 | 1,021 | 3 | 1,735 | 4 | 1,647 | 4 | 2,295 | 5 | 2,144 | 5 | 2,660 | 5 | 490 | 1 | 1,281 | 3 | 1,902 | 4 | 1,013 | 3 | 19,234 | 1,602 | |  |
| j | 8,756 | | 21 | 9,114 | 21 | 7,960 | 20 | 9,058 | 21 | 9,095 | 21 | 9,683 | 21 | 9,471 | 21 | 9,875 | 20 | 7,234 | 19 | 9,059 | 22 | 9,403 | 21 | 7,962 | 20 | 106,670 | 8,889 | |  |
| o | 501 | | 1 | 526 | 1 | 380 | 1 | 500 | 1 | 563 | 1 | 614 | 1 | 549 | 1 | 670 | 1 | 398 | 1 | 507 | 1 | 542 | 1 | 468 | 1 | 6,218 | 518 | |  |
| p | 836 | | 2 | 881 | 2 | 806 | 2 | 1,073 | 2 | 913 | 2 | 1,015 | 2 | 998 | 2 | 1,195 | 2 | 686 | 2 | 801 | 2 | 984 | 2 | 781 | 2 | 10,969 | 914 | |  |
| s | 3 | | 0 | 7 | 0 | 2 | 0 | 4 | 0 | 7 | 0 | 6 | 0 | 7 | 0 | 8 | 0 | 4 | 0 | 6 | 0 | 7 | 0 | 6 | 0 | 67 | 5 | |  |
| u | 4,221 | | 10 | 4,276 | 10 | 2,849 | 7 | 4,375 | 10 | 4,784 | 11 | 5,443 | 12 | 5,233 | 12 | 7,583 | 16 | 2,724 | 7 | 3,872 | 9 | 4,558 | 10 | 3,367 | 8 | 53,285 | 4,440 | |  |
| x | 161 | | 0 | 164 | 0 | 107 | 0 | 143 | 0 | 198 | 0 | 215 | 1 | 207 | 0 | 254 | 1 | 114 | 0 | 150 | 0 | 149 | 1 | 132 | 0 | 1,994 | 166 | |  |
| **Total** | **42,647** | |  | **43,298** |  | **39,850** |  | **43,669** |  | **43,948** |  | **46,179** |  | **45,480** |  | **49,060** |  | **37,941** |  | **42,377** |  | **44,333** |  | **40,283** |  | **519,065** | **43,255** | |  |
|  | | | | | | | | | | | | | | | | | | | | | | | | | | |  |  |  |
| = | | Exactly equal to the reference annotation | | | | | | | | | | | | | | | | | | | | | | | | |  |  | |
| c | | Contained in the reference annotation | | | | | | | | | | | | | | | | | | | | | | | | |  |  | |
| e | | Possible pre-mRNA molecule | | | | | | | | | | | | | | | | | | | | | | | | |  |  | |
| i | | An exon falling into an intron of the reference | | | | | | | | | | | | | | | | | | | | | | | | |  |  | |
| j | | New isoforms | | | | | | | | | | | | | | | | | | | | | | | | |  |  | |
| o | | Unknown, generic overlap with reference | | | | | | | | | | | | | | | | | | | | | | | | |  |  | |
| p | | Possible polymerase run-on fragment | | | | | | | | | | | | | | | | | | | | | | | | |  |  | |
| s | | An intron of the transfag overlapping a reference intron on the opposite strand | | | | | | | | | | | | | | | | | | | | | | | | |  |  | |
| u | | Unknown, intergenic transcript | | | | | | | | | | | | | | | | | | | | | | | | |  |  | |
| x | | Exonic overlap with reference on the opposite strand | | | | | | | | | | | | | | | | | | | | | | | | |  |  | |
| TA | | Transcript Assembly | | | | | | | | | | | | | | | | | | | | | | | | |  |  | |
