# Supplementary material for: Differences in Muscle Transcriptome among Pigs Phenotypically Extreme for Fatty Acid Composition
Source: PLoS One. 2014 Jun 13;9(6):e99720. doi: 10.1371/journal.pone.0099720 (PMC4057286; doi:10.1371/journal.pone.0099720)
Supplement: Table S3 — Description of the repetitive elements identified in the intergenic transcripts of the swine muscle transcriptome. (DOCX) [file pone.0099720.s004.docx]

| **Table S3.** Description of the repetitive elements identified in the intergenic transcripts of the swine muscle transcriptome. | | | | | | | | | |  |
| --- | --- | --- | --- | --- | --- | --- | --- | --- | --- | --- |
|  |  |  | | |  | |  | |  | |
|  | | **Number of elements** | | | **Length occupied (bp)** | | **Percentage sequence** | |  | |
| **SINEs:** | | 54,081 | | | 10,653,056 | | 14.12 | |  | |
|  | Alu/B1 | 0 | | | 0 | | 0.00 | |  | |
|  | MIRs | 10,755 | | | 1,520,033 | | 2.01 | |  | |
| **LINEs:** | | 24,147 | | | 11,260,471 | | 14.92 | |  | |
|  | LINE1 | 17,049 | | | 9,425,600 | | 12.49 | |  | |
|  | LINE2 | 6,161 | | | 1,607,095 | | 2.13 | |  | |
|  | L3/CR1 | 752 | | | 181,472 | | 0.24 | |  | |
|  | RTE | 182 | | | 45,909 | | 0.06 | |  | |
| **LTR elements:** | | 7,062 | | | 2,567,345 | | 3.40 | |  | |
|  | ERVL | 1,695 | | | 695,439 | | 0.92 | |  | |
|  | ERVL-MaLRs | 3,462 | | | 1,143,189 | | 1.51 | |  | |
|  | ERV_classI | 1,581 | | | 631,945 | | 0.84 | |  | |
|  | ERV_classII | 87 | | | 41,151 | | 0.05 | |  | |
| **DNA elements:** | | 7,275 | | | 1,531,597 | | 2.03 | |  | |
|  | hAT-Charlie | 4,583 | | | 873,939 | | 1.16 | |  | |
|  | TcMar-Tigger | 1,307 | | | 389,915 | | 0.52 | |  | |
| **Unclassified:** | | 39 | | | 8,158 | | 0.01 | |  | |
| **Total interespersed repeats** | |  | | | 26,020,627 | | 34.48 | |  | |
| Small RNA: |  | 113 | | | 10,536 | | 0.01 | |  | |
| Satellites: |  | 8 | | | 5,395 | | 0.01 | |  | |
| Simple repeats: |  | 12,310 | | | 497,650 | | 0.66 | |  | |
| Low complexity: |  | 10,336 | | | 400,389 | | 0.53 | |  | |
|  |  |  | |  | |  | |  | |  |
| Total length: 75,471,156 bp | |  | |  | |  | |  | |  |
| GC level: 43.88 % |  |  | |  | |  | |  | |  |
| Bases masked: 26,929,199 bp (35.68%) | | |  |  | |  | |  | |  |
