# Supplementary material for: Differences in Muscle Transcriptome among Pigs Phenotypically Extreme for Fatty Acid Composition
Source: PLoS One. 2014 Jun 13;9(6):e99720. doi: 10.1371/journal.pone.0099720 (PMC4057286; doi:10.1371/journal.pone.0099720)
Supplement: Table S5 — Differentially-expressed genes identified among extreme groups (High and Low) for fatty acid composition in muscle. (DOCX) [file pone.0099720.s006.docx]

| **Table S5.** Differentially-expressed genes identified between extreme groups (High and Low) for fatty acid composition in muscle. | | | | | | | | | | | | | |
| --- | --- | --- | --- | --- | --- | --- | --- | --- | --- | --- | --- | --- | --- |
|  | |  |  | |  | |  | |  | |  | | |
| **Overlapping DE genes from DESeq and EdgeR analyses** | **Associated ID** | | | **Fold Change** | | ***P*-value** | | ***Q*-value** | | **Mean Reads Low** | | **Mean Reads High** | |
| *ENSSSCG00000026478* | *PADI2* | | | -11.89 | | 3.60E-27 | | 4.76E-24 | | 326,7 | | | 27,5 |
| *ENSSSCG00000006932* | *CLCA4* | | | -5.26 | | 1.09E-06 | | 1.15E-04 | | 29,8 | | | 5,7 |
| *ENSSSCG00000006035* | *ANGPT1* | | | -3.38 | | 4.36E-04 | | 1.39E-02 | | 46,0 | | | 13,6 |
| *ENSSSCG00000020756* | *FAM101A* | | | -3.26 | | 5.64E-05 | | 3.39E-03 | | 78,7 | | | 24,2 |
| *ENSSSCG00000013513* | *PLIN5* | | | -3.13 | | 1.27E-09 | | 2.58E-07 | | 284,9 | | | 91,1 |
| *ENSSSCG00000000577* | *GYS2* | | | -3.11 | | 7.39E-05 | | 4.16E-03 | | 19,6 | | | 6,3 |
| *ENSSSCG00000010554* | *SCD* | | | -3.08 | | 1.29E-12 | | 4.27E-10 | | 660,0 | | | 214,3 |
| *ENSSSCG00000024306* | *UBCH5B* | | | -2.89 | | 9.46E-04 | | 2.28E-02 | | 37,2 | | | 12,9 |
| *ENSSSCG00000024342* | *AQP4* | | | -2.86 | | 1.02E-09 | | 2.25E-07 | | 368,1 | | | 128,7 |
| *ENSSSCG00000029099* | *ERGIC3* | | | -2.69 | | 2.12E-04 | | 9.51E-03 | | 19,7 | | | 7,3 |
| *ENSSSCG00000002292* | *PLEKHH1* | | | -2.68 | | 6.41E-04 | | 1.77E-02 | | 41,9 | | | 15,6 |
| *ENSSSCG00000006245* | *SDR16C5* | | | -2.60 | | 6.13E-05 | | 3.60E-03 | | 38,5 | | | 14,8 |
| *ENSSSCG00000017947* | *ACADVL* | | | -2.33 | | 5.32E-05 | | 3.27E-03 | | 512,5 | | | 219,8 |
| *ENSSSCG00000017501* | *PNMT* | | | -2.27 | | 1.19E-03 | | 2.77E-02 | | 79,5 | | | 35,1 |
| *ENSSSCG00000010635* | *ZDHHC6* | | | -2.26 | | 4.43E-03 | | 6.97E-02 | | 39,3 | | | 17,4 |
| *ENSSSCG00000011848* | *TFRC* | | | -2.24 | | 1.05E-06 | | 1.15E-04 | | 2848,1 | | | 1269,2 |
| *ENSSSCG00000008959* | *CXCL2* | | | -2.21 | | 2.73E-05 | | 2.12E-03 | | 186,8 | | | 84,4 |
| *ENSSSCG00000016958* | *PIK3R1* | | | -2.19 | | 2.25E-05 | | 1.80E-03 | | 77,9 | | | 35,6 |
| *ENSSSCG00000009079* | *INTU* | | | -2.16 | | 8.64E-03 | | 1.14E-01 | | 30,4 | | | 14,1 |
| *ENSSSCG00000010348* | *CDHR1* | | | -2.13 | | 6.16E-03 | | 8.93E-02 | | 75,0 | | | 35,2 |
| *ENSSSCG00000023287* | *MYL6B* | | | -2.10 | | 2.57E-03 | | 4.90E-02 | | 1224,3 | | | 583,3 |
| *ENSSSCG00000006003* | *MAL2* | | | -2.08 | | 5.05E-04 | | 1.53E-02 | | 43,0 | | | 20,7 |
| *ENSSSCG00000011755* | *NCEH1* | | | -2.08 | | 8.57E-05 | | 4.72E-03 | | 166,8 | | | 80,2 |
| *ENSSSCG00000007288* | *MYH7B* | | | -2.07 | | 1.05E-04 | | 5.33E-03 | | 3034,6 | | | 1468,2 |
| *ENSSSCG00000029304* | *STEAP3* | | | -2.03 | | 2.58E-04 | | 1.10E-02 | | 225,8 | | | 111,4 |
| *ENSSSCG00000029558* | *EXTL1* | | | -1.99 | | 3.18E-03 | | 5.57E-02 | | 202,3 | | | 101,6 |
| *ENSSSCG00000004248* | *PLN* | | | -1.97 | | 9.52E-05 | | 5.04E-03 | | 4710,5 | | | 2385,2 |
| *ENSSSCG00000007909* | *ABAT* | | | -1.97 | | 3.48E-03 | | 5.82E-02 | | 36,8 | | | 18,7 |
| *ENSSSCG00000009074* | *C4ORF29* | | | -1.94 | | 1.43E-04 | | 7.01E-03 | | 411,3 | | | 212,1 |
| *ENSSSCG00000024681* | *TECRL* | | | -1.92 | | 1.19E-03 | | 2.77E-02 | | 496,8 | | | 258,6 |
| *ENSSSCG00000023806* | *LRRN1* | | | -1.89 | | 2.63E-03 | | 4.97E-02 | | 108,8 | | | 57,5 |
| *ENSSSCG00000025527* | *FABP3* | | | -1.87 | | 5.23E-04 | | 1.57E-02 | | 589,7 | | | 315,8 |
| *ENSSSCG00000005449* | *PTPN3* | | | -1.86 | | 1.67E-03 | | 3.51E-02 | | 282,7 | | | 152,2 |
| *ENSSSCG00000029058* | *C14H10orf116* | | | -1.85 | | 8.75E-04 | | 2.12E-02 | | 173,7 | | | 93,6 |
| *ENSSSCG00000023548* | *GSTCD* | | | -1.83 | | 1.12E-03 | | 2.65E-02 | | 135,5 | | | 74,2 |
| *ENSSSCG00000002383* | *FOS* | | | -1.81 | | 2.63E-07 | | 3.48E-05 | | 4424,6 | | | 2441,5 |
| *ENSSSCG00000010992* | *AQP7* | | | -1.80 | | 4.42E-03 | | 6.97E-02 | | 162,3 | | | 90,0 |
| *ENSSSCG00000003546* | *FABP3* | | | -1.77 | | 1.48E-03 | | 3.16E-02 | | 703,8 | | | 397,4 |
| *ENSSSCG00000017498* | *PPP1R1B* | | | -1.77 | | 1.37E-03 | | 3.02E-02 | | 83,9 | | | 47,5 |
| *ENSSSCG00000007227* | *ID1* | | | -1.76 | | 6.32E-03 | | 8.96E-02 | | 155,7 | | | 88,5 |
| *ENSSSCG00000024954* | *FGF1* | | | -1.75 | | 4.15E-04 | | 1.39E-02 | | 519,5 | | | 297,0 |
| *ENSSSCG00000010537* | *GOT1* | | | -1.73 | | 2.88E-04 | | 1.16E-02 | | 5639,9 | | | 3259,7 |
| *ENSSSCG00000007094* | *ENSSSCG00000007094* | | | -1.73 | | 9.07E-03 | | 1.18E-01 | | 172,2 | | | 99,7 |
| *ENSSSCG00000010456* | *PANK1* | | | -1.73 | | 8.85E-03 | | 1.16E-01 | | 137,2 | | | 79,5 |
| *ENSSSCG00000015886* | *ITGB6* | | | -1.72 | | 1.48E-03 | | 3.16E-02 | | 619,4 | | | 360,1 |
| *ENSSSCG00000010631* | *GPAT* | | | -1.72 | | 2.23E-03 | | 4.50E-02 | | 1032,8 | | | 600,5 |
| *ENSSSCG00000004602* | *TEX9* | | | -1.71 | | 2.30E-04 | | 1.01E-02 | | 593,0 | | | 346,1 |
| *ENSSSCG00000005437* | *KLF2* | | | -1.71 | | 4.29E-05 | | 2.88E-03 | | 504,3 | | | 295,0 |
| *ENSSSCG00000011218* | *SLC4A7* | | | -1.71 | | 1.37E-03 | | 3.02E-02 | | 659,9 | | | 386,7 |
| *ENSSSCG00000009534* | *BIVM* | | | -1.71 | | 2.68E-04 | | 1.13E-02 | | 1372,1 | | | 804,4 |
| *ENSSSCG00000026078* | *GLCE* | | | -1.67 | | 4.35E-04 | | 1.39E-02 | | 568,7 | | | 339,6 |
| *ENSSSCG00000009865* | *TBX3* | | | -1.67 | | 3.77E-03 | | 6.19E-02 | | 330,4 | | | 197,6 |
| *ENSSSCG00000011557* | *CIDE-C* | | | -1.67 | | 6.17E-04 | | 1.74E-02 | | 128,3 | | | 77,0 |
| *ENSSSCG00000001844* | *PLIN1* | | | -1.65 | | 8.36E-06 | | 7.13E-04 | | 183,2 | | | 111,1 |
| *ENSSSCG00000021281* | *TRIP10* | | | -1.65 | | 5.57E-04 | | 1.60E-02 | | 165,0 | | | 100,0 |
| *ENSSSCG00000006769* | *MCT1* | | | -1.65 | | 3.79E-04 | | 1.33E-02 | | 1607,4 | | | 975,4 |
| *ENSSSCG00000027030* | *BDKRB2* | | | -1.65 | | 9.97E-03 | | 1.27E-01 | | 45,2 | | | 27,4 |
| *ENSSSCG00000027344* | *ATP2A2* | | | -1.64 | | 3.44E-04 | | 1.25E-02 | | 5961,3 | | | 3633,3 |
| *ENSSSCG00000030197* | *CD2AP* | | | -1.62 | | 6.39E-03 | | 8.99E-02 | | 357,1 | | | 219,8 |
| *ENSSSCG00000007077* | *ESF1* | | | -1.62 | | 6.56E-03 | | 9.13E-02 | | 617,1 | | | 380,3 |
| *ENSSSCG00000004215* | *KIAA0408* | | | -1.60 | | 2.76E-03 | | 5.11E-02 | | 632,9 | | | 395,4 |
| *ENSSSCG00000014827* | *PLEKHB1* | | | -1.59 | | 2.93E-03 | | 5.28E-02 | | 203,7 | | | 127,8 |
| *ENSSSCG00000006682* | *POLR3GL* | | | -1.59 | | 4.92E-03 | | 7.48E-02 | | 1105,2 | | | 693,7 |
| *ENSSSCG00000002831* | *IRX3* | | | -1.59 | | 2.06E-03 | | 4.26E-02 | | 427,3 | | | 268,5 |
| *ENSSSCG00000011477* | *ACOX2* | | | -1.59 | | 4.61E-03 | | 7.17E-02 | | 427,5 | | | 268,7 |
| *ENSSSCG00000017759* | *ALDOA* | | | -1.58 | | 4.53E-05 | | 2.92E-03 | | 230,8 | | | 145,9 |
| *ENSSSCG00000016991* | *DUSP1* | | | -1.57 | | 6.99E-05 | | 4.02E-03 | | 5735,8 | | | 3657,6 |
| *ENSSSCG00000010893* | *FH* | | | -1.57 | | 5.69E-03 | | 8.36E-02 | | 360,5 | | | 230,1 |
| *ENSSSCG00000006857* | *COL11A1* | | | -1.56 | | 3.14E-03 | | 5.54E-02 | | 95,6 | | | 61,2 |
| *ENSSSCG00000010429* | *PRKG1* | | | -1.56 | | 4.71E-03 | | 7.25E-02 | | 552,3 | | | 353,8 |
| *ENSSSCG00000023264* | *IDH1* | | | -1.55 | | 3.44E-03 | | 5.80E-02 | | 426,0 | | | 274,3 |
| *ENSSSCG00000001723* | *PAF-AH* | | | -1.55 | | 6.02E-04 | | 1.71E-02 | | 1600,8 | | | 1031,9 |
| *ENSSSCG00000000555* | *ITPR2* | | | -1.54 | | 6.26E-03 | | 8.94E-02 | | 290,2 | | | 187,9 |
| *ENSSSCG00000014889* | *NDUFC2* | | | -1.53 | | 3.72E-03 | | 6.15E-02 | | 211,3 | | | 138,2 |
| *ENSSSCG00000008504* | *CRIM1* | | | -1.50 | | 2.72E-03 | | 5.07E-02 | | 652,4 | | | 434,1 |
| *ENSSSCG00000022099* | *TP53INP2* | | | -1.49 | | 5.63E-03 | | 8.31E-02 | | 4778,1 | | | 3200,2 |
| *ENSSSCG00000029683* | *ENSSSCG00000029683* | | | -1.48 | | 8.05E-04 | | 2.03E-02 | | 425,6 | | | 287,0 |
| *ENSSSCG00000006866* | *DBT* | | | -1.45 | | 6.03E-03 | | 8.81E-02 | | 783,1 | | | 539,1 |
| *ENSSSCG00000003909* | *PIK3R3* | | | -1.44 | | 9.10E-03 | | 1.18E-01 | | 722,9 | | | 500,3 |
| *ENSSSCG00000008237* | *RETSAT* | | | -1.43 | | 4.04E-03 | | 6.56E-02 | | 843,3 | | | 591,4 |
| *ENSSSCG00000010532* | *LOXL4* | | | -1.36 | | 8.23E-04 | | 2.05E-02 | | 224,7 | | | 165,4 |
| *ENSSSCG00000010678* | *NANOS1* | | | 1.78 | | 4.54E-06 | | 4.41E-04 | | 249,7 | | | 443,5 |
| *ENSSSCG00000015854* | *OCA2* | | | 1.79 | | 5.21E-03 | | 7.78E-02 | | 40,8 | | | 73,0 |
| *ENSSSCG00000010850* | *ENAH* | | | 1.80 | | 3.14E-07 | | 3.96E-05 | | 1645,3 | | | 2961,6 |
| *ENSSSCG00000016983* | *STC2* | | | 1.80 | | 8.05E-04 | | 2.03E-02 | | 291,6 | | | 525,9 |
| *ENSSSCG00000015986* | *HOXD1* | | | 1.81 | | 1.86E-04 | | 8.63E-03 | | 98,5 | | | 178,3 |
| *ENSSSCG00000012142* | *AP1S2* | | | 1.83 | | 5.36E-04 | | 1.58E-02 | | 44,7 | | | 81,6 |
| *ENSSSCG00000021205* | *MST4* | | | 1.87 | | 2.04E-04 | | 9.29E-03 | | 92,1 | | | 172,2 |
| *ENSSSCG00000002279* | *GPX2* | | | 1.92 | | 3.44E-03 | | 5.80E-02 | | 19,0 | | | 36,4 |
| *ENSSSCG00000001097* | *FAM65B* | | | 1.93 | | 3.19E-05 | | 2.41E-03 | | 297,0 | | | 571,9 |
| *ENSSSCG00000010464* | *PPP1R3C* | | | 1.94 | | 3.73E-05 | | 2.74E-03 | | 4374,7 | | | 8479,0 |
| *ENSSSCG00000014362* | *HBEGF* | | | 1.95 | | 1.85E-07 | | 2.72E-05 | | 1155,8 | | | 2258,5 |
| *ENSSSCG00000011265* | *CMYA1* | | | 1.96 | | 2.37E-03 | | 4.71E-02 | | 62973,1 | | | 123374,9 |
| *ENSSSCG00000013374* | *KCNC1* | | | 1.96 | | 7.69E-04 | | 2.01E-02 | | 98,1 | | | 192,3 |
| *ENSSSCG00000027428* | *ENHO* | | | 1.96 | | 9.45E-06 | | 7.81E-04 | | 618,6 | | | 1215,5 |
| *ENSSSCG00000030165* | *MAFF* | | | 2.00 | | 3.83E-04 | | 1.33E-02 | | 427,6 | | | 854,5 |
| *ENSSSCG00000010974* | *CNTFR* | | | 2.02 | | 4.84E-06 | | 4.41E-04 | | 738,2 | | | 1494,8 |
| *ENSSSCG00000021104* | *Pseudogene* | | | 2.08 | | 3.35E-03 | | 5.80E-02 | | 66,6 | | | 138,2 |
| *ENSSSCG00000028384* | *RND3* | | | 2.08 | | 1.11E-08 | | 2.10E-06 | | 720,8 | | | 1498,2 |
| *ENSSSCG00000017251* | *SOX9* | | | 2.10 | | 7.57E-03 | | 1.02E-01 | | 42,6 | | | 89,2 |
| *ENSSSCG00000020701* | *TTC9* | | | 2.11 | | 2.87E-08 | | 4.47E-06 | | 1191,0 | | | 2512,7 |
| *ENSSSCG00000010219* | *ARID5B* | | | 2.12 | | 6.90E-06 | | 6.08E-04 | | 2323,1 | | | 4936,4 |
| *ENSSSCG00000008973* | *NAAA* | | | 2.14 | | 7.61E-04 | | 2.01E-02 | | 58,0 | | | 124,1 |
| *ENSSSCG00000015331* | *PON2* | | | 2.15 | | 6.23E-03 | | 8.94E-02 | | 51,9 | | | 111,4 |
| *ENSSSCG00000023085* | *STAC* | | | 2.26 | | 1.01E-04 | | 5.26E-03 | | 52,8 | | | 119,3 |
| *ENSSSCG00000015340* | *ASNS* | | | 2.32 | | 2.75E-10 | | 7.27E-08 | | 401,2 | | | 931,5 |
| *ENSSSCG00000012967* | *FOSL1* | | | 2.35 | | 3.01E-04 | | 1.16E-02 | | 74,9 | | | 176,2 |
| *ENSSSCG00000008348* | *PLEK* | | | 2.38 | | 4.18E-05 | | 2.88E-03 | | 82,6 | | | 196,7 |
| *ENSSSCG00000028282* | *SLC1A4* | | | 2.47 | | 4.98E-03 | | 7.53E-02 | | 9,9 | | | 24,5 |
| *ENSSSCG00000022913* | *SLPI* | | | 2.49 | | 9.95E-07 | | 1.14E-04 | | 86,7 | | | 216,1 |
| *ENSSSCG00000024428* | *CHRNA9* | | | 2.57 | | 1.48E-03 | | 3.16E-02 | | 33,9 | | | 87,2 |
| *ENSSSCG00000021569* | *MMP25* | | | 2.62 | | 1.46E-03 | | 3.16E-02 | | 11,2 | | | 29,4 |
| *ENSSSCG00000015589* | *VASH2* | | | 2.63 | | 9.13E-07 | | 1.10E-04 | | 47,5 | | | 124,8 |
| *ENSSSCG00000015595* | *ATF3* | | | 2.66 | | 2.24E-13 | | 8.46E-11 | | 1718,3 | | | 4566,1 |
| *ENSSSCG00000029066* | *IDI1* | | | 2.71 | | 2.97E-04 | | 1.16E-02 | | 31,9 | | | 86,4 |
| *ENSSSCG00000010555* | *HIF1AN* | | | 2.77 | | 2.07E-11 | | 6.08E-09 | | 80,2 | | | 221,8 |
| *ENSSSCG00000004302* | *C6ORF165* | | | 2.77 | | 7.25E-04 | | 1.96E-02 | | 20,3 | | | 56,3 |
| *ENSSSCG00000026686* | *PDZD9* | | | 2.78 | | 1.92E-08 | | 3.17E-06 | | 161,3 | | | 447,8 |
| *ENSSSCG00000022060* | *RASSF9* | | | 2.78 | | 9.25E-03 | | 1.19E-01 | | 16,3 | | | 45,2 |
| *ENSSSCG00000025136* | *ACTN3* | | | 2.83 | | 4.79E-10 | | 1.15E-07 | | 10044,0 | | | 28453,2 |
| *ENSSSCG00000022059* | *RPL27A* | | | 2.84 | | 4.96E-04 | | 1.53E-02 | | 17,0 | | | 48,1 |
| *ENSSSCG00000015797* | *SORBS2* | | | 3.13 | | 4.78E-05 | | 3.01E-03 | | 51,8 | | | 162,4 |
| *ENSSSCG00000028568* | *HSPB1* | | | 3.35 | | 1.87E-14 | | 8.24E-12 | | 231,4 | | | 775,3 |
| *ENSSSCG00000027368* | *CTSF* | | | 3.49 | | 2.49E-07 | | 3.47E-05 | | 30,4 | | | 105,9 |
| *ENSSSCG00000004754* | *CHAC1* | | | 3.52 | | 8.53E-04 | | 2.09E-02 | | 10,7 | | | 37,5 |
| *ENSSSCG00000003647* | *FHL3* | | | 3.79 | | 4.35E-05 | | 2.88E-03 | | 15,8 | | | 59,9 |
| *ENSSSCG00000011640* | *TF* | | | 3.99 | | 2.45E-03 | | 4.79E-02 | | 8,9 | | | 35,3 |
| *ENSSSCG00000024482* | *HSPB1* | | | 4.25 | | 7.64E-24 | | 6.74E-21 | | 218,9 | | | 929,3 |
| *ENSSSCG00000005385* | *NOR-1* | | | 8.04 | | 1.65E-44 | | 4.36E-41 | | 266,6 | | | 2144,1 |
| *ENSSSCG00000008948* | *ALB* | | | 8.69 | | 1.13E-18 | | 7.47E-16 | | 32,0 | | | 278,0 |
| *ENSSSCG00000012961* | *BANF1* | | | 11.15 | | 7.87E-16 | | 4.16E-13 | | 9,3 | | | 103,5 |
