# Supplementary material for: Correction: Differences in Muscle Transcriptome among Pigs Phenotypically Extreme for Fatty Acid Composition
Source: PLoS One. 2014 Jul 21;9(7):e103668. doi: 10.1371/journal.pone.0103668 (PMC4105474; doi:10.1371/journal.pone.0103668)
Supplement: File S2 — Republished, corrected article. (PDF) [file pone.0103668.s002.pdf]

OPEN ACCESS Freely available online

PLOS ONE

Differences in Muscle Transcriptome among Pigs Phenotypically Extreme for Fatty Acid Composition

Anna Puig-Oliveras[1,2]*, Yuliaxis Ramayo-Caldas[3], Jordi Corominas[1,2], Jordi Estellé[3,4,5], Dafne Pérez-Montarelo[6], Nicholas J. Hudson[7], Joaquim Casellas[2,8], Josep M. Folch[1,2], Maria Ballester[1,2]

1 Departament de Genètica Animal, Centre de Recerca en Agrigenòmica (CRAG), Bellaterra, Spain, **2** Departament de Ciència Animal i dels Aliments, Universitat Autònoma de Barcelona (UAB), Bellaterra, Spain, **3** Génétique Animale et Biologie Intégrative UMR1313 (GABI), Institut National de la Recherche Agronomique (INRA), Jouy-en-Josas, France, **4** Génétique Animale et Biologie Intégrative UMR1313 (GABI), AgroParisTech, Jouy-en-Josas, France, **5** Laboratoire de Radiobiologie et Etude du Génome (LREG), Commissariat à l'énergie atomique et aux énergies alternatives (CEA), Jouy-en-Josas, France, **6** Departamento de Genética Animal, Instituto Nacional de Investigación y Tecnología Agraria y Alimentaria (INIA), Madrid, Spain, **7** Computational and Systems Biology, Commonwealth Scientific and Industrial Research Organisation (CSIRO) Animal, Food and Health SciencesQLD, Brisbane, Australia, **8** Departament de Genètica i Millora Animal, Institut de Recerca i Tecnologies Agroalimentàries (IRTA), Lleida, Spain

Abstract

Background: Besides having an impact on human health, the porcine muscle fatty acid profile determines meat quality and taste. The RNA-Seq technologies allowed us to explore the pig muscle transcriptome with an unprecedented detail. The aim of this study was to identify differentially-expressed genes between two groups of 6 sows belonging to an Iberian × Landrace backcross with extreme phenotypes according to FA profile.

Results: We sequenced the muscle transcriptome acquiring 787.5 M of 75 bp paired-end reads. About 85.1% of reads were mapped to the reference genome. Of the total reads, 79.1% were located in exons, 6.0% in introns and 14.9% in intergenic regions, indicating expressed regions not annotated in the reference genome. We identified a 34.5% of the intergenic regions as interspersed repetitive regions. We predicted a total of 2,372 putative proteins. Pathway analysis with 131 differentially-expressed genes revealed that the most statistically-significant metabolic pathways were related with lipid metabolism. Moreover, 18 of the differentially-expressed genes were located in genomic regions associated with IMF composition in an independent GWAS study in the same genetic background. Thus, our results indicate that the lipid metabolism of FAs is differently modulated when the FA composition in muscle differs. For instance, a high content of PUFA may reduce FA and glucose uptake resulting in an inhibition of the lipogenesis. These results are consistent with previous studies of our group analysing the liver and the adipose tissue transcriptomes providing a view of each of the main organs involved in lipid metabolism.

Conclusions: The results obtained in the muscle transcriptome analysis increase the knowledge of the gene regulation of IMF deposition, FA profile and meat quality, in terms of taste and nutritional value. Besides, our results may be important in terms of human health.

Citation: Puig-Oliveras A, Ramayo-Caldas Y, Corominas J, Estellé J, Pérez-Montarelo D, et al. (2014) Differences in Muscle Transcriptome among Pigs Phenotypically Extreme for Fatty Acid Composition. PLoS ONE 9(6): e99720. doi:10.1371/journal.pone.0099720

Editor: Hervé Guillou, INRA, France

Received December 23, 2013; **Accepted** May 19, 2014; **Published** June 13, 2014

Copyright: © 2014 Puig-Oliveras et al. This is an open-access article distributed under the terms of the Creative Commons Attribution License, which permits unrestricted use, distribution, and reproduction in any medium, provided the original author and source are credited.

Funding: This work has been funded by MICINN project AGL2011-29821-C02 (Ministerio de Economía y Competitividad) and by the Innovation Consolider-Ingenio 2010 Program (CSD2007-00036). A. Puig-Oliveras was funded by a Personal Investigador en Formación (PIF) PhD grant from the Universitat Autònoma de Barcelona (458-01-1/2011). J. Corominas was funded by a Formación de Personal Investigador (FPI) PhD grant from Spanish Ministerio de Educación (BES-2009-018223), Y. Ramayo by a Formación del Profesorado Universitario (FPU) PhD grant (AP2008-01450). The funders had no role in study design, data collection and analysis, decision to publish, or preparation of the manuscript.

Competing Interests: The authors have declared that no competing interests exist.

* E-mail: anna.puig@cragenomica.es

Introduction

High-throughput sequencing technologies are rapidly evolving and its application to transcriptome analysis (RNA-Seq), with the adapted bioinformatic tools, allow the exploration of the transcriptome in an unprecedented manner in terms of accuracy and data insight [1]. In addition, RNA-Seq technology is useful, not only to detect variation in gene expression patterns, but also to identify new isoforms, splicing events, and different promoter and polyadenylation signal usage. Currently, only a few RNA-Seq studies have been conducted in livestock species such as pigs [2–6].

According to the Food and Agriculture Organization (FAO) [7], pork is the major source of meat intake by human, accounting for the 43% of the consumed meat worldwide. The taste and the quality of the cooked and the cured meat products depend on the oxidative stability of the muscle which is related to the fatty acid (FA) composition [8,9]. Furthermore, it is well known that genetic and environmental factors such as diet, are responsible for FA composition variation [10]. Besides its influence on meat taste, the FA composition in muscle has taken additional importance due to their nutritional value and human health-related benefits [11,12], particularly for its effects on human diseases such as cancers,

coronary heart diseases and atherosclerosis [11]. It has been reported that omega-3 FAs, such as α-linolenic acid (C18:3 n-3), are associated with the reduction of low density lipoprotein (LDL) cholesterol and blood triacylglycerols, as well as with the modulation of immune functions and inflammatory processes [13,14]. Artificial selection to increase meat production in pigs has caused a reduction of intramuscular fat (IMF) and changes in meat FA composition in some breeds. Hereby, there is an increasing interest in the pork industry on producing meat products with a higher IMF content and with a healthier FA profile, while maintaining a reduced amount of backfat [15].

In a recent genome-wide association study (GWAS) [16], genomic regions associated with the IMF (*Longissimus dorsi*) FA composition were identified in a backcross population (BC1_LD; 25% Iberian and 75% Landrace). A combined linkage QTL scan and GWAS performed in the same backcross revealed significant pleiotropic regions with effects on both IMF and backfat tissues [17,18]. Moreover, the transcriptome of the other two major organs regulating lipid metabolism, liver and adipose tissue (backfat), have been studied using RNA-Seq in the BC1_LD animals [4,5]. In these studies, a shift towards the oxidation of FAs in liver [4] and an inhibition of *de novo* lipogenesis in adipose tissue [5] was observed in animals with higher content of polyunsaturated FA (PUFA). Since the adipose and liver tissues have previously been analysed using animals belonging to the same population, with the addition of the muscle transcriptome we aim to have a more complete view of the genetic regulation of lipid metabolism in pigs [4,5]. The goal of the current study was to identify differentially-expressed genes and pathways in the *Longissimus dorsi* muscle of Iberian × Landrace backcrossed pigs with extreme phenotypes for muscle FA profile to better understand the differences in this meat quality trait.

Results

Phenotypic differences among the analysed animals

In a previous work [4], a Principal Component Analysis (PCA) was performed to select animals of an Iberian × Landrace backcross (BC1_LD) with extreme phenotypes for IMF FA composition. Using the same classification and the first principal component, six females belonging to the extreme High (H) group and six from the Low (L) group were selected for muscle RNA-Seq analysis. Animal selection considered the parental genetic diversity according to the pedigree. Significant statistical differences (*P*-value <0.05) were identified between the H and L groups in 18 out of 26 evaluated traits (Table 1). The H group had, in comparison to the L group, a higher content of PUFA including linolenic (C18:2 n-6), α-linolenic, eicosadienoic (C20:2 n-6), eicosatrienoic (C20:3 n-6) and arachidonic (C20:4 n-6) FAs. Conversely, the L group had a higher content of monounsaturated FA (MUFA) like palmitoleic (C16:1 n-7) and oleic (C18:1 n-9) FAs and saturated FAs (SFA) such as myristic (C14:0) and palmitic (C16:0) FAs. The two groups of pigs did not differ significantly in either IMF content or backfat thickness.

Transcriptome analysis of swine muscle tissue

As described above, the *Longissimus dorsi* (LD) muscle transcriptome was sequenced in twelve sows (H = 6, L = 6) with extreme phenotypes for intramuscular FA composition. A total amount of 787.5 M of 75 bp paired-end reads were acquired from the RNA-Seq experiment. Sequence alignment was performed against the reference pig genome (Sscrofa10.2) by using Tophat [19]. About 85.1% (76.5%–86.6%) of reads were mapped to the reference genome, of which 14.5% (12.4%–16.1%) did not map to unique genomic locations. A total of 85.1% (84.0%–87.6%) of the mapped reads correspond to annotated genes, 79.1% (77.5%–84.1%) of them were located in exons and 6.0% (3.6%–6.8%) in introns. The remaining 14.9% (12.4%–16.0%) of reads mapped to intergenic regions, indicating that they were not annotated in the reference genome (Table S1).

The transcripts generated when assembling the short reads with Cufflinks [20] resulted in a mean of 43,255 transcripts expressed in muscle (Table S2). Transcripts were classified in different categories, being the most abundant the exonic transcripts (60.4%), the putative new isoforms (20.5%) and the intergenic transcripts (10.1%) (Table S2). A total of 9,887 new isoforms were identified corresponding to 9,805 known genes.

Transposable elements identification and novel coding gene discovery

The percentage of interspersed repeats identified with the Repeat Masker [21] in the intergenic transcripts was about 34.5%. Moreover, SINEs and LINEs were the most abundant repetitive elements identified (14.1% and 14.9%, respectively) (Table S3).

With the aim of improving the current porcine genome annotation, we took into account the intergenic transcripts identified with cufflinks (a mean of 4,440 transcripts) to determine whether these transcripts potentially codified for proteins. A total of 2,372 putative proteins were predicted by Augustus [22] corresponding to non-annotated transcripts of the Sscrofa10.2 genome assembly version. Among the 2,372 novel predicted proteins, only 1,406 (59.2%) had at least one orthologous gene identified with BLASTP option of Blast2GO, representing a total of 577 known genes (Table S4) [23]. These proteins corresponded to: 720 *Sus scrofa*, 17 *Homo sapiens* and 247 *Bos taurus in silico* predicted protein, and 476 *Sus scrofa*, 933 *Homo sapiens*, and 403 *Bos taurus* known proteins. The pig species was the only one showing a higher percentage of computationally predicted protein (60.2%) in comparison to known proteins (39.8%).

Moreover, 918 of the predicted novel proteins were successfully annotated with Blast2GO [23]. To summarize the functional annotation, a GO Slim analysis was performed. The most relevant molecular functions identified were "protein binding" (25%), "ion binding" (19%), "nucleic acid binding" (17%), "small molecular binding" (10%) and, interestingly, "lipid binding" (2%). These new transcripts were mainly involved in the following biological processes: "primary metabolic process" (9%), "cellular metabolic process" (9%), "macromolecule metabolic process" (8%) and "regulation of biological process" (7%). Using the Enzime code and KEGG, the main metabolic pathways represented were the "phosphatidylinositol signalling system" (12 sequences), "inositol phosphate metabolism" (11) and the "pyrimidine (9) and purine (8) metabolism".

Differential gene expression analysis

A high correlation (r = 0.98, *P*-value $<2.2\times10^{-16}$) between H and L groups in the mean gene expression was found, showing that most of the genes had a similar behaviour. A total of 11,945 transcripts were used to perform the differential expression analysis after filtering. Using EdgeR program [24], 314 genes were identified as significantly differentially expressed between H and L groups. Whereas, employing DESeq [25], 208 genes were detected. Figure 1 shows the *P*-value distribution and how among the transcripts accepted as differentially expressed the selected cut-off of *P*-value <0.01 is clearly departing from the expected *P*-value (equivalent to a FDR ≤0.12).

A total of 131 genes (Table S5) overlapping in both analyses were selected as differentially expressed between H and L groups

Table 1. Mean comparison between High and Low groups (six animals per group) of the traits included in the principal component analysis (PCA).

Characters	H Mean	L Mean	Significance	*P*-value
**Carcass quality**				
Carcass weight (CW) (kg)	66.22±10.52	70.50±7.91	NS	4.44×10^{-1}
Ham weight (HW) (kg)	18.79±2.32	18.97±2.41	NS	8.96×10^{-1}
Shoulder weight (SW) (kg)	6.6±0.95	6.32±0.97	NS	6.16×10^{-1}
Intramuscular fat (IMF) (%)	1.94±0.65	1.69±0.64	NS	5.25×10^{-1}
**Fatty acids in intramuscular fat**				
Saturated FA [a]				
Myristic acid (C14:0)	1.11±0.12	1.28±0.12	*	$3,08\times10^{-2}$
Palmitic acid (C16:0)	21.29±0.57	24.16±0.54	***	4.35×10^{-6}
Heptadecanoic acid (C17:0)	0.35±0.06	0.20±0.03	***	3.09×10^{-4}
Stearic acid (C18:0)	13.50±0.94	14.16±1.11	NS	2.91×10^{-1}
Arachidic acid (C20:0)	0.25±0.09	0.23±0.05	NS	5.83×10^{-1}
Monounsaturated FA [a]				
Palmitoleic acid (C16:1 n-7)	2.33±0.30	2.97±0.41	*	1.03×10^{-2}
Heptadecenoic acid (C17:1)	0.33±0.08	0.22±0.05	*	2.04×10^{-2}
Oleic acid (C18:1 n-9)	36.78±3.10	42.77±1.07	**	1.18×10^{-3}
Octadecenoic acid (C18:1 n-7)	3.85±0.20	4.14±0.27	NS	6.29×10^{-2}
Eicosenoic acid (C20:1 n-9)	0.82±0.13	0.82±0.07	NS	9.95×10^{-1}
Polyunsaturated FA [a]				
Linoleic acid (C18:2 n-6)	13.70±1.30	6.83±0.40	***	2.14×10^{-7}
α-Linolenic acid (C18:3 n-3)	1.14±0.42	0.47±0.07	**	3.23×10^{-3}
Eicosadienoic acid (C20:2 n-6)	0.61±0.16	0.38±0.05	**	8.39×10^{-3}
Eicosatrienoic acid (C20:3 n-6)	0.42±0.17	0.15±0.02	**	3.58×10^{-3}
Arachidonic acid (C20:4 n-6)	2.79±1.26	0.76±0.18	**	2.96×10^{-3}
Metabolic ratios				
Average Chain Length (ACL)	17.44±0.02	17.37±0.02	***	2.41×10^{-7}
Saturated FA (SFA)	36.49±1.08	40.02±1.28	***	4.20×10^{-4}
Monounsaturated FA (MUFA)	44.49±2.90	51.21±1.41	***	4.67×10^{-4}
Polyunsaturated FA (PUFA)	18.67±2.75	8.59±0.53	***	4.95×10^{-6}
Peroxidability index (PI)	30.92±6.66	13.53±1.02	***	8.60×10^{-5}
Double-bond index (DBI)	0.44±0.08	0.19±0.01	***	2.85×10^{-5}
Unsaturated index (UI)	0.89±0.06	0.70±0.01	***	1.82×10^{-5}

NS: *P*-value >0.05; * *P*-value <0.05; ** *P*-value <0.01; *** *P*-value <0.001
[a]The percentage of each FA, relative to the total FA
doi:10.1371/journal.pone.0099720.t001

and, thereafter, used for pathway analysis (Figure 2). Fifty genes had a higher expression and 81 a lower expression in the H group (in comparison with L group). Remarkably, eighteen (*CLCA4, ANGPT1, PLEKHH1, SDR16C5, PIK3R1, INTU, MAL2, NCEH1, PLN, C4orf29, FABP3, TBX3, MCT1, ESF1, POLR3GL, DBT, C6orf165* and *CHAC1*) of the 131 genes were also present in the annotated QTL intervals of a GWAS study for the IMF FA profile performed in the same population [16]. Three of these genes (*PIK3R1*, *NCEH1* and *FABP3*) have been directly related with lipid metabolism [26], being clear candidate genes to study the genetic contribution of IMF FA composition. Intriguingly, only two (*C6orf165* and *CHAC1*) of the 18 genes were over-expressed in the H group. Moreover, two of the differentially-expressed genes in muscle (*AQP7* and *FOS*) were also identified as differentially expressed in liver [4], and seventeen of them (*AQP4, SCD, PLEKHB1, CTSF, CIDEC, ALDOC, CXCL2, KIAA0408, SLPI, ALB, C14H10orf116, ITPR2, TRIP10, BANF1, HIF1AN, CHAC1* and *FHL3*) were identified as differentially expressed in adipose tissue [5]. In addition, three of the differentially-expressed genes in our analysis (*ATF3, ENAH* and *SLPI*) were also identified in a muscle microarray study of extreme animals for FA composition from the same backcross [27]. Other genes such as *DNAJA4, ANKRD1, MYH10* and *TNFRSF12A* were also common, but they were only detected by the DESeq program [25] in the RNA-Seq data.

Functional analysis

With the aim of having a more complete functional view of our differentially-expressed genes in the H and L groups, we used Babelomics [28] and Ingenuity Pathways Analysis [29] programs, who have related capabilities but use different databases. The top canonical pathways overrepresented according to IPA were related with nitric oxide signalling in the cardiovascular system

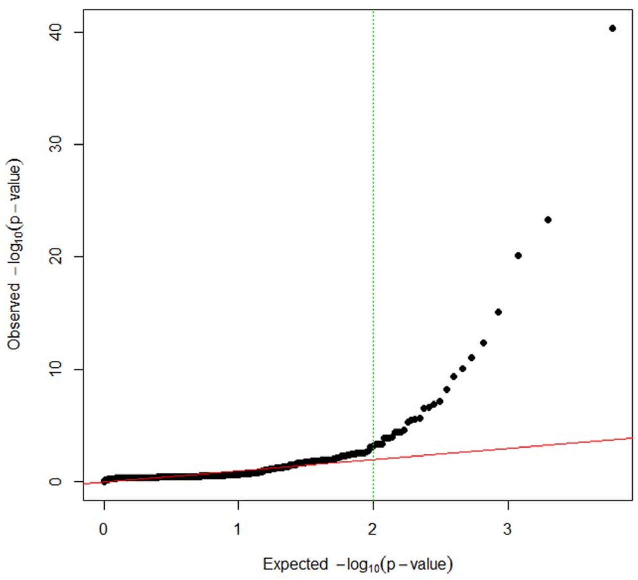

Figure 1. Q-Q plot representing the DESeq [25] ***P*-value distribution of the differentially expression analysis.** The expected distribution of the *P*-values is indicated with a red line, whereas black points represent the observed distribution. The selected cut-off is represented with a green discontinuous line (-log10 (*P*-value) >2).
doi:10.1371/journal.pone.0099720.g001

(7 genes, *P*-value $=7.75\times10^{-7}$) and endothelial nitric oxide synthase signalling (*eNOS*, 8 genes, *P*-value $=2.34\times10^{-6}$). On the other hand, using Babelomics we observed an overrepresentation of lipids and lipoproteins metabolism (6 genes, *P*-value $=1.64\times10^{-3}$), and also the peroxisome proliferator-activated receptors (*PPAR*, 4 genes, *P*-value $=7.25\times10^{-4}$) and the insulin (5 genes, *P*-value $=1.00\times10^{-3}$) signalling and the hemostasis (7 genes, *P*-value $=1.64\times10^{-3}$) pathway (Table 2).

Among the top molecular and cellular functions significantly overrepresented when comparing H relative to L groups with Babelomics, we identified the response to organic substance (18 genes, *P*-value $=3.8\times10^{-7}$), the muscle organ development (5 genes, *P*-value $=3.8\times10^{-5}$), the energy derivation by oxidation of organic compounds (5 genes, *P*-value $=1.0\times10^{-4}$) and the response to hormone stimulus (9 genes, *P*-value $=2.6\times10^{-4}$). Whereas with IPA, the most relevant functions were involved in lipid metabolism (30 genes, *P*-value $=1.04\times10^{-6}$), molecular transport (36 genes, *P*-value $=1.04\times10^{-06}$), small molecule biochemistry (47 genes, *P*-value $=1.04\times10^{-6}$), cell death and survival (38 genes, *P*-value $=1.55\times10^{-6}$), carbohydrate metabolism (30 genes, *P*-value $=2.25\times10^{-6}$), energy production (10 genes, *P*-value $=5.8\times10^{-5}$) and skeletal and muscular system development and function (23 genes, *P*-value $=2.47\times10^{-4}$) (Table S6).

Among the related specific functions for lipid metabolism, the top molecular functions identified with IPA were the oxidation (*ACADVL, ACOX2, FABP3, PLIN1, PLIN5, PON2, SCD*; *P*-value $=3.59\times10^{-4}$), accumulation (*ACADVL, AQP7, FH, IDH1, PLIN1, PON2, RETSAT, SCD*; *P*-value $=7.90\times10^{-04}$), synthesis

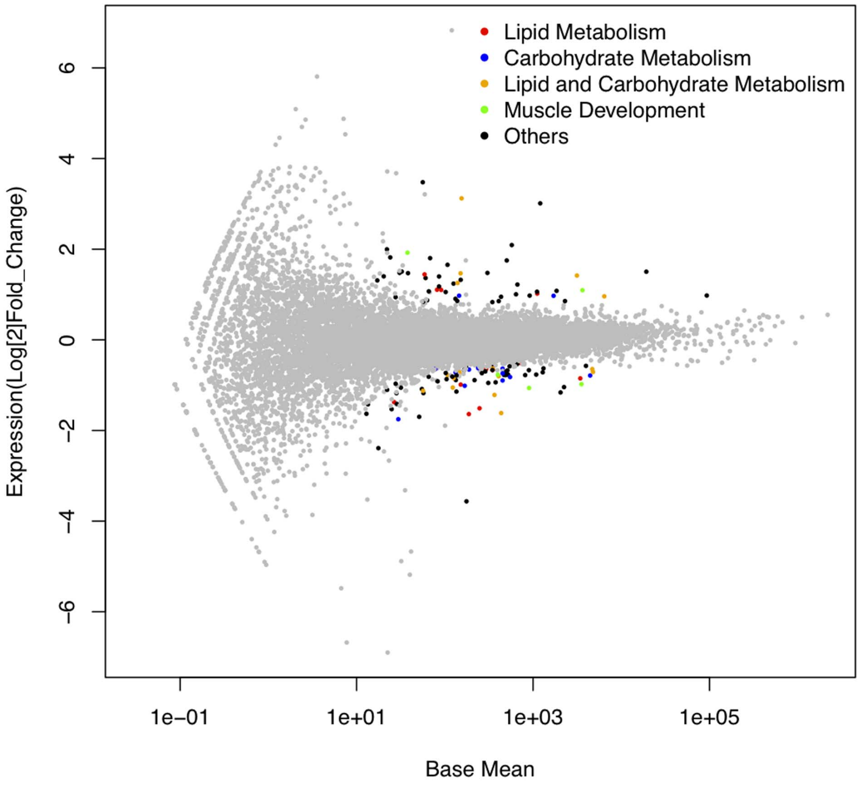

Figure 2. Plot of the 131 differentially-expressed genes identified between the two groups High and Low. X-axis values correspond to base mean expression values and y-axis values are the log2(fold change). The colour for the differentially-expressed genes is related to their function in lipid metabolism (red), carbohydrate metabolism (blue), both lipid and carbohydrate metabolism (orange), muscle development (green) or others (black).
doi:10.1371/journal.pone.0099720.g002

Table 2. Summary of the most significantly-overrepresented pathways of the differentially-expressed genes in muscle between High and Low groups for fatty acid composition traits.

**Babelomics**			**IPA**		
**Category**	**Genes**	***P*-value**	**Category**	**Genes**	***P*-value**
Metabolism of Lipids and Lipoproteins	*SCD, ACADVL, ACOX2, IDH1, IDI1, ALB*	5.12×10^{-4}	Nitric Oxid Signalling in the Cardiovascular System	*BDKRB2, PIK3R3, PRKG1, PLN, ITPR2, PIK3R1, ATP2A2*	7.75×10^{-7}
Alanine, Aspartate and Glutamate Metabolism	*ABAT, GOT1, ASNS*	6.38×10^{-4}	eNOS Signalling	*BDK4B2, PIK3R3, AQP7, PRKG1, ITPR2, PIK3R1, CHRNA9, AQP4*	2.34×10^{-6}
PPAR Signalling Pathway	*ACOX2, AQP7, FABP3, SCD*	7.25×10^{-4}	Clathrin-mediated Endocytosis Signalling	*PIK3R3, ALB, CD2AP, TF, PIK3R1, TFRC, ITGB6, FGF1*	2.95×10^{-5}
Insulin Signalling Pathway	*GYS2, PIK3R1, TRIP10, PIK3R3, PPP1R3C*	1.00×10^{-3}	*ILK* Signalling	*PIK3R3, FOS, RND3, PIK3R1, ITGB6, MYL6B, MYH7B, ACTN3*	2.95×10^{-5}
Hemostasis	*ANGPT1, PIK3R1, ALDOA, ITPR2, PLEK, TF, ALB*	1.64×10^{-3}	*CXCR4* Signalling	*PIK3R3, FOS, RND3, ITPR2, PIK3R1, MYL6B*	4.79×10^{-4}

doi:10.1371/journal.pone.0099720.t002

(*ACADVL, ACOX2, ALB, BDKRB2, CNTFR, FABP3, FGF1, FOS, IDI1, PIK3R1, PLIN1, PON2, SCD*; *P*-value $=2.28\times10^{-3}$), concentration (*DUSP1, EXTL1, FABP3, FOS, IDH1, NCEH1, PLIN1, PON2, PPP1R3C, SCD*; *P*-value $=2.48\times10^{-5}$) and homeostasis (*ACADVL, FABP3, GOT1, NCEH1, PIK3R1, PLIN1, SCD*; *P*-value $=1.96\times10^{-4}$) of lipids (Table S7). Other related pathways were identified such as concentration of bile acids (*ALB, ATF3, SCD*; *P*-value $=3.87\times10^{-4}$), obesity (*ABAT, AQP7 ARID5B, ATF3, DESP1, HBEGF, IDH1, PLIN1, RETSAT, SCD*; *P*-value $=5.76\times10^{-4}$), and insulin resistance (*ACOX2, ALB, AQP7, ATP2A2, FGF1, PIK3R1, PON2, PPP1R3C, SCD*; *P*-value $=5.41\times10^{-3}$) and sensitivity (*FABP3, HIF1AN, PIK3R1, SCD*; *P*-value $=7.39\times10^{-3}$). In addition, interesting functions such as heart disease, blood pressure, glucose tolerance, synthesis of carbohydrate and glucose metabolism disorder, biogenesis of cholesterol, differentiation of muscle cells and adiposity were also identified (Table S7).

Finally, a total of nine direct and nine indirect networks were obtained with IPA (Table S8). The top direct network was associated with cell death and survival, cellular development, connective tissue development and function (Figure 3). It showed a score of 55 and contained 29 molecules (Table S8). The top indirect network was related to metabolic disease, lipid metabolism and molecular transport (Figure S1). A total of 21 molecules were associated to this network having a score of 36 (Table S8).

Discussion

To date, muscle transcriptome analyses concerning meat quality in swine have mainly been conducted using microarrays [3,27,30–32]. Compared with microarrays, RNA-Seq enables to determine the transcript abundance with a larger dynamic range of expression levels, it is not limited by the available genomic sequencing information during microarray production and can provide information about new isoforms. However, the main RNA-Seq drawback when compared with microarrays is that the analysis relies on the current pig genome assembly (in this study 10.2), in which interesting genes involved in lipid metabolism are still incorrectly annotated or not present. Therefore, the improvement of the annotation is transcendental for further RNA-Seq studies.

Muscle transcriptome description

In the present study, using RNA-Seq analysis we were able to map a high percentage of reads to the current pig genome assembly (Sscrofa10.2). Our percentage of mapped reads (85.1%) was similar to the described in the pig adipose tissue transcriptome (80%–87%) [5] performed with the Sscrofa10.2 annotation version, however it was higher than the percentage found in the pig muscle transcriptome (64,8%) [6] performed with the Sscrofa9.2 version or the pig liver transcriptome (71.4%–77.7%) [4] using the Sscrofa9.61 annotation version. The high amount of transcripts mapping to intergenic regions and the novel coding gene discovery, showing a higher percentage of computationally predicted proteins (60.2%) versus known proteins (39.8%) in pig in comparison to other species such as bovine and human, reinforces the need to improve the current pig annotation. Similar results were shown in the porcine liver [4] and adipose tissue [5] transcriptomes, in which the 86.0% and 62.5% of the novel proteins identified respectively were computationally-predicted. As expected, the major overlap of predicted novel proteins was between muscle and adipose tissue [5] because unlike the liver and gonads [2,4], both analyses were performed using the most recent annotation of the genome. Hence, a total of 40% novel predicted proteins in the muscle tissue transcriptome have also been found in adipose tissue, either realised with the Sscrofa10.2. Of the 2,372 predicted novel proteins, 972 were validated *in silico* being present in at least one of the three tissues compared [2,4,5]. Interestingly, 36 of the novel predicted proteins were also identified in four different tissues (liver [4], gonads [2], adipose [5], and muscle tissue) (Figure 4). When analysing the main metabolic pathways for the novel transcripts identified, the "phosphatidylinositol signalling system" and "inositol phosphate metabolism" were among the most represented categories. The phosphatidylinositol signalling system plays a critical role in the regulation of diverse processes such as muscle contraction, cell secretion, cell growth and differentiation [33]. Moreover, phosphatidylinositol is an essential component of the lipid membrane, where the total amount of phospholipids remains fairly constant, or increases little, as the animal increases in fatness [9,34]. Not surprisingly, these pathways were also identified when analyzing the adipose tissue novel transcripts [5]. Interestingly, the phosphatidylinositol signalling was also found within the most significantly overrepresented pathways in animals differing in FA composition in an study using microarrays [35]. Finally, we detected a high percentage (34.5%) of new repetitive elements present in the porcine genome. This result was similar to those obtained in adipose tissue (36%) using the Sscrofa10.2 genome annotation, but higher than those obtained in gonads (7.3%) and liver (approximately 5.8–7.3%)

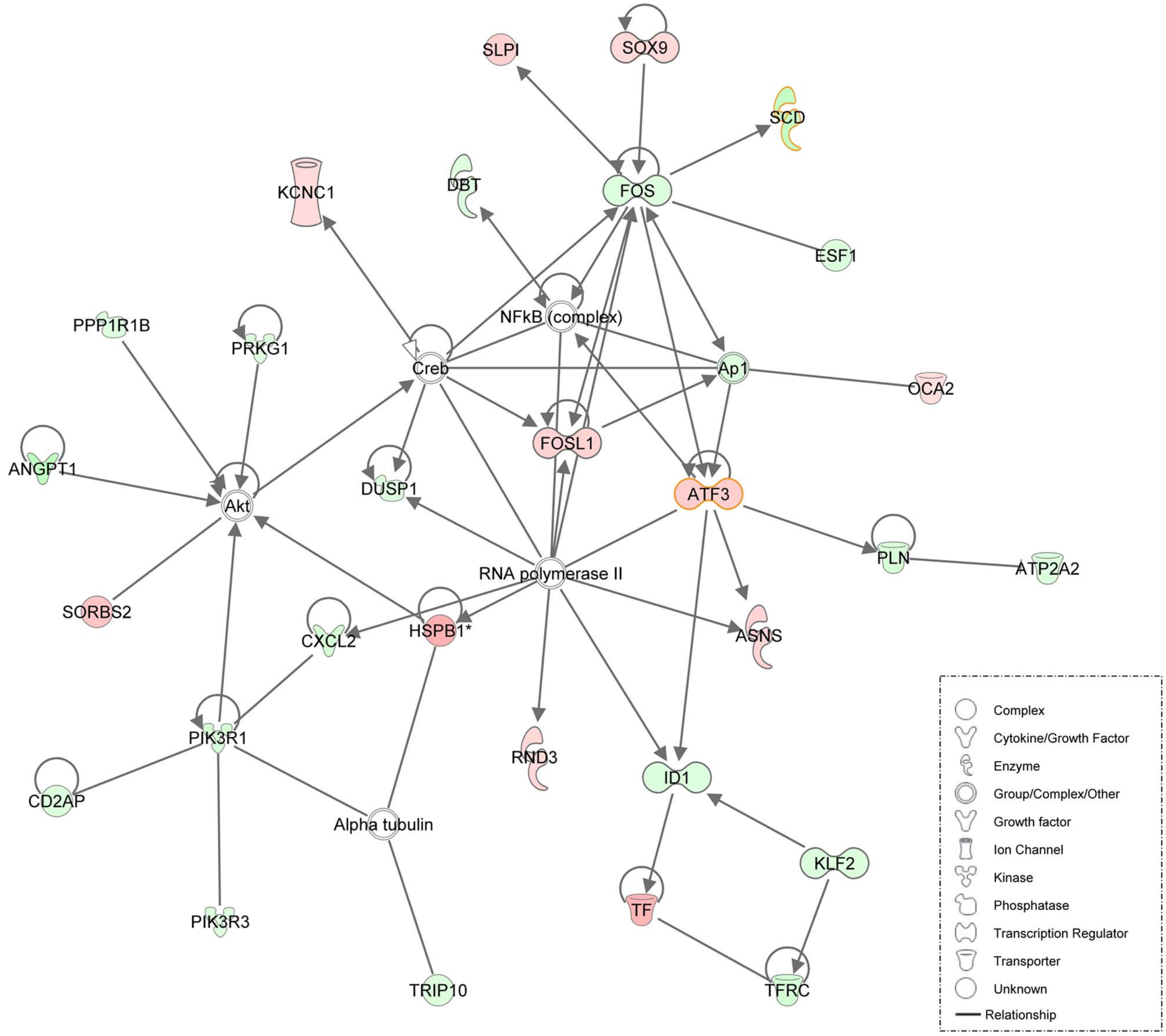

© 2000-2013 Ingenuity Systems, Inc. All rights reserved.

Figure 3. Network (direct, score 55) generated by IPA of 35 focus genes corresponding to the cell death and survival, cellular development, connective tissue and function pathways. Node colours indicate gene expression, being the red nodes higher-expressed genes and the green nodes lower-expressed genes in the H group relative to the L group. Colour intensity is related to the degree of expression. Node shapes indicate the biological function of the protein.
doi:10.1371/journal.pone.0099720.g003

that used an older version of the pig genome assembly [2,4,5]. This higher content of repetitive elements can be explained by the improvement of the current assembly (Sscrofa10.2) of the pig genome being the repetitive regions the most difficult to assemble [36].

Differential Expression analysis

Apart from describing the transcriptome of the *Longissimus dorsi* muscle, this study aimed to identify genes that can be implicated in determining the phenotypic differences of animals with extreme IMF FA composition belonging to an Iberian x Landrace backcross (BC1_LD). Iberian pigs are a local Mediterranean breed, and in comparison with Landrace, they have an extreme trend to obesity, with a higher IMF content and a strongest development of backfat tissue [8]. In contrast to the Iberian pigs, commercial breeds such as Landrace have suffered a strong selection towards a lean meat content, muscularity and enhanced reproduction [37]. Moreover, these two breeds are extreme for FA composition, showing the Iberian pigs a higher content of SFA and MUFA (specially C16 and C18:1) and the Landrace pigs a higher content of PUFA [8,38]. In our study, animals belonging to L group had a higher content of SFA and MUFA similarly to the Iberian pigs, whereas animals from H group had higher content of PUFA, as observed in the Landrace animals. Thus, this animal material suits very well to studies aiming at identifying the molecular factors influencing the FA metabolism in pigs.

For the differential-expression analysis we intersected the two lists of genes, obtained by DEseq [25] and EdgeR [24], to obtain a

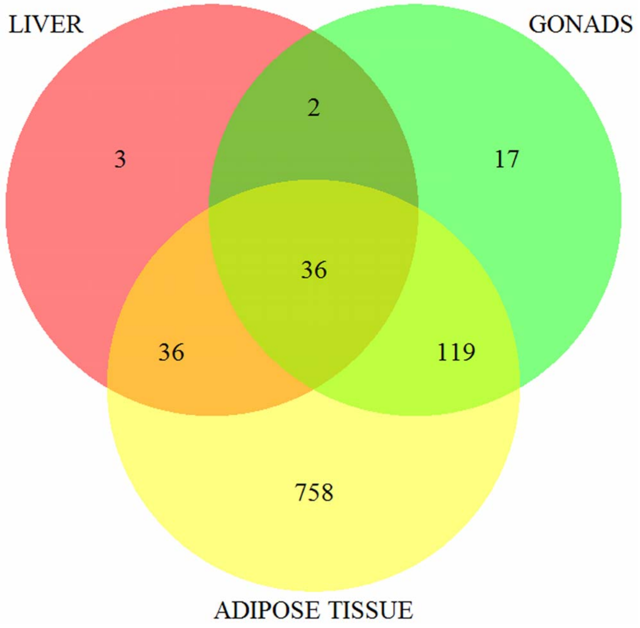

Figure 4. Venn diagram of the novel predicted proteins expressed in muscle, liver [4], gonads [2] and adipose tissue [5].
doi:10.1371/journal.pone.0099720.g004

single list in order to avoid false positives [39]. For the same reason we used a strict *P*-value ≤0.01, based on the Q-Q plot and equivalent to a FDR ≤0.12, and a fold change ≥1.2 as used in the adipose transcriptome analysis [5,40]. We identified a lower number of differentially-expressed genes with DESeq (208 genes) when compared to EdgeR (314 genes) what is in accordance with the observations reported by Soneson & Delorenzi [40]. DESeq uses conservative default settings and performs well when outliers are introduced, having a better false discovery rate (FDR) control for large sample sizes than EdgeR [40]. Notice that some relevant genes identified using microarrays in the muscle transcriptome of animals extreme for FA metabolism [27,35] such as *ACACA, FABP4* or *PPARGC1A* remained incorrectly or non-annotated in the Sscrofa10.2 annotation version. On the other hand, interesting genes detected in our RNA-Seq study that may determine differences in FA composition in muscle such as *ChREBP, GYS2, PLIN1, PLIN5* and *AQP7* could not be detected in microarray studies since probes for these genes were not included.

Differentially modulated metabolic pathways between groups

Among the top canonical pathways overrepresented between both groups of animals, we found hemostasis, nitric oxide (NO), metabolism of lipids and lipoproteins and PPAR and insulin signalling pathways (Table 2). Remarkably, most of the genes represented in these pathways were down-expressed in the H group. When compared with a previous study using microarrays of animals of BC1_LD population [27], the insulin and the calcium signalling, the regulation of the cytoskeleton, the focal adhesion dynamics, the leukocyte accumulation and cardiomyopathies-related pathways (Table S7) were found in common. Interestingly, our analysis identified other relevant pathways related to lipid metabolism, PPAR and NO signalling. On the other hand, most of the main overrepresented pathways identified in our study were also present in Duroc animals displaying divergent MUFA and PUFA fatty acids percentages (Table 2) [35], thus supporting a relevant role of these metabolic pathways in determining intramuscular FA composition. However, a feedback loop in which FA composition modifies these metabolic pathways that in turn cause a change in FA composition cannot be ruled out as we described below (i.e. differences in C16:1 n-7 FA or PUFA). Besides, in our analysis two other interesting pathways were found: (i) the clathrin-mediated endocytosis signalling, which is used for molecules such as low density lipoproteins, transferrins or growth factors and (ii) the C-X-C chemokine receptor type 4 (CXCR4) signalling, involved in the endocytosis of the glucose transporter protein 4 (GLUT4), specially in myocytes [41].

In the following sections a detailed explanation of differentially-expressed genes belonging to each of the main overrepresented pathways will be discussed.

- **NO and insulin signalling pathways.** The skeletal muscle is a target organ for the insulin-induced glucose uptake and for the maintenance of glucose homeostasis in blood [42]. Insulin acts in the carbohydrate metabolism facilitating the glucose diffusion into adipose and muscle cells via glucose transporter proteins (GLUT) and stimulates FA synthesis and the storage of triglycerides by the esterification of glycerol phosphate. Notably, the C16:1 n-7 FA, observed to be decreased in the H group (Table 1), can act as a lipokine that jointly with the expression of the *peroxisome proliferator-activated receptor gamma* (*PPAR-γ*) can strongly stimulate the muscle insulin action [43,44]. Interestingly, *PPAR-γ* and *solute carrier family 2, member 4* (*SLC2A4*; also called *GLUT4*) were identified as over-expressed in the L group animals when using EdgeR program. Supporting these results, Cánovas *et al.* [35] identified a higher expression of *myocyte enhancer factor 2A* (*MEF2A*) gene which upregulates *GLUT4* in Duroc animals having a higher MUFA and SFA content. Furthermore, insulin stimulates eNOS, the enzyme responsible for synthesizing NO by calcium-independent phosphorylation via phosphoinositide 3-kinases (PI3 kinases) and the downstream effector serine/threonine kinase (Akt) [45]. NO is a signalling molecule synthesized from L-arginine that plays an important role in regulating energy metabolism in mammals [46]. It has been reported that a chronic exposure of NO may decrease whole-body energy metabolism, increasing the adiposity and obesity [46]. For instance, *PI3 kinases*, down-expressed in the H group, have been reported to be necessary for the insulin-stimulated glucose uptake and glycogen synthesis, meanwhile, *Akt* regulates cell growth and metabolism and it is involved in glucose transport and lipogenesis (Figure 4) [16,47]. In the same direction, the *glycogen synthase* (*GYS*) gene was down-expressed in animals belonging to the H group (Figure S1), what might be a downstream effect of the Akt pathway [48]. Thus, the *GYS* inhibition may decrease the synthesis of glycogen necessary for glucose storage.
- **PPAR and metabolism of lipids and lipoproteins pathways.** In concordance with the low glucose-uptake that seems to occur in the H group of animals, we observed a down-expression of lipogenic genes most probably due to the lack of activation of carbohydrate responsive-element binding protein (*ChREBP*) [49]. The *stearoyl-CoA desaturase* (*SCD*) gene (Table S5) is responsible for the biosynthesis of MUFA from SFA, and its deficiency has been associated with lean mice [50]. Furthermore, polymorphisms in *SCD* gene have been strongly associated with FA composition in pigs and cows [12,51–53]. It has been suggested that an inhibition of this enzyme produces an increase in fatty acid oxidation through the inhibition of *acetyl-CoA carboxylase* (*ACACA*), regulated via

ChREBP, and *de novo* lipogenesis [50,54]. Interestingly, the *SCD* gene was identified as down-expressed in the adipose tissue of animals with higher content of PUFA in the Iberian x Landrace crossbred [5] and over-expressed in animals with higher IMF accumulation [31,35]. Our results support the hypothesis of Corominas *et al.* [5], that suggested that higher PUFA content in the H group suppresses the *ChREBP* gene function in a LXR-dependent manner inhibiting glycolytic and lipogenic genes. Although not present in the overlapping list (Table S5), the *ChREBP* gene was identified as down-expressed in the H group for EdgeR program.

Another gene whose disruption is associated with lean mice and was also down-expressed in H group is the *perilipin* [55]. Perilipins modulate the hydrolysis of triglycerides by *hormone-sensitive lipase* (*LIPE*) [56]. Specifically, *perilipin 5* (*PLIN5*) may play a role of "master lipolytic regulator" in muscle, and its over-expression can increase lipid droplet size and triacylglycerol storage [57]. We also identified the lipid transporter *Fatty acid binding protein 3, muscle and heart* (*FABP3*) and the *Aquaporins* (*AQP4* and *AQP7*) as down-expressed in the H group (Table S4). *FABP3* is more expressed in the skeletal muscle than in other tissues and participates in FA uptake and cytosolic transport, having a high binding affinity for palmitic, oleic and stearic acids. Furthermore, *FABP3* acts as a transcription factor in the nucleus for the control of lipid-mediated transcriptional programs via nuclear hormone receptors or other transcription factors that respond to lipids [58]. This gene has also been found in a genomic region significantly associated with FA composition in a GWAS performed in the Iberian x Landrace cross, being a clear candidate to explain the differences in FA composition observed between the two groups of animals [16]. Besides, it has been suggested as a candidate gene for the control of IMF deposition as it was identified as over-expressed in animals with higher IMF content [59]. The *Aquaporins* are modulated by the PI3K/Akt signalling and they are involved in glycerol uptake, particularly *AQP4* is localized in muscle fibers and it is important for energy supply in the skeletal muscle [46,60,61]. The *AQP7* which is higher expressed in fat tissue was also identified in the liver transcriptome study as being also down-expressed in animals with a higher content of PUFA [4,61]. Another differentially-expressed gene between the two groups of animals was the *very long-chain specific acyl-CoA dehydrogenase* gene (*ACADVL*), a *PPAR* target gene which was down-expressed in the H group. This gene catalyzes the first step of the mitochondrial FA β-oxidation pathway, mainly in muscle, having preference for C16:0, C16:1, C18:0 and C18:1 [62,63]. Moreover, *ACADVL* deficiency in humans produced a defective oxidation of oleic FA and knock-out mice for *ACADVL* fed in high-fat diet had a decrease in whole body fat content [64]. Overall, these results agree with a previous study in which the transcriptome of two groups of Duroc pigs with different IMF composition was analysed using microarrays and concluded that the IMF accumulation in animals having more IMF, MUFA and SFA may result from a balance between uptake, synthesis and degradation of triglycerides [35].

- **Hemostasis.** Alterations in fat metabolism play a role in the development of cardiovascular disease. Not surprisingly, our data set revealed several differentially-expressed transcripts which could be classified as potential regulators of hemostasis (Table S2). For instance, the *angiopoietin-1* (*ANGPT1*) gene which has been reported to increase vessel formation causing an enhanced glucose uptake and also the glycogen and lipid synthesis [65] was over-expressed in the L group and present in a QTL interval of the GWAS for IMF FA profile in the same population (Table S5) [16]. Furthermore, and consistent with our results, the angiogenesis promoted by ANGPT1 has been reported to increase NO production accompanied by an activation of the Akt and the nuclear factor kappa-light-chain-enhancer of activated B cells (NF-κB) signalling pathways [66,67] (Figure 4). Accordingly, the *hypoxia-inducible factor-1, alpha subunit inhibitor* (*HIF1AN*), which is regulated through the NF-κB inflammatory pathway and serves as an oxygen sensor regulating heart's oxygen supply, was down-expressed in the L group (Table S5). Thus, the L group animals having more SFA and MUFA content may have boosted the angiogenesis and improved the inflammatory response through the activation of the *ANGPT1* gene. A decreased essential PUFA content may lead to a proinflammatory eicosanoids synthesis and vasoconstrictors activation as has been reported in other studies [68]. In this direction, an over-expression of genes encoding for the inositol 1,4,5-triphosphate receptor 2 (ITPR2) protein which activates the release of Ca(2+) in the vessels acting as vasoconstrictor and aldosterone A (ALDOA) which increases blood pressure when activated by angiotensin [69,70] was observed in L group.

Pig lipid metabolism affected by intramuscular FA composition

In general, our results show that differences in FA composition may influence the lipid metabolism determining the phenotypic variation observed between the two groups of animals. In previous studies of our group, we observed that in liver [4], a high content of PUFA (H group phenotype) shifted the metabolism towards the FA oxidation; meanwhile, in adipose tissue [5] inhibited lipogenesis. Accordingly, in other studies analyzing the muscle transcriptome using microarrays a favored FA oxidation and a reduced fatty acid uptake, lipogenesis and triacylglycerol synthesis was generally observed in the group with higher intramuscular PUFA content [27,35]. In our RNA-Seq study in muscle we observed an inhibition of glucose uptake and lipogenesis in the H group, which would produce a decrease in the triglyceride storage. Noteworthy, in adipose and muscle transcriptome analysis, the *albumin* (*ALB*) gene was identified as over-expressed in animals having a higher content of PUFA (H group) [5]. The ALB is a long chain FA transporter that enhances FA mobilization affecting cellular uptake and also plays an antioxidant function in plasma. In adipose tissue, we hypothesized that ALB was supplying the FFAs used for the oxidation in liver in pre-slaughtering fasting conditions [5]. In the same direction, our results suggested that in muscle there is also an increased input of FFAs from blood and adipose tissue in order to fulfil the high-energy requirements in the H group. We hypothesize that animals having a high content of SFA and MUFA such as the Iberian pig, which is a rustic and slow-growing breed, may have an enhanced adaptation to fasting thanks to their high availability of muscle energy stores. Thus, selection towards a fast growth in commercial pigs such as Landrace, may have affected the ability to adapt to food disposal fluctuations [71].

Implications

In conclusion, the genes identified here as differentially-expressed between extreme animals, the pathways and the gene networks, contribute to understand the differences in gene regulation between the two groups differing in the muscle FA

composition. The functional analysis showed a different regulation of the lipid metabolism between groups, being more prone either to lipolisis or to lipogenesis depending on their FA composition. Moreover, the enrichment analysis showed that muscle plays a key role in energy metabolism, mainly in glucose and lipid metabolism, observing that animals having more PUFA, had a shift of the metabolism towards the lipolisis and also a lower glucose uptake. There are also evidences that the joint metabolism of the liver, adipose tissue and muscle may have an integrated role in determining the final FA composition of muscle. Therefore, the study of the muscle transcriptome may provide clues to decipher the genetic basis of meat quality traits. Moreover, this study may be of high relevance since FA composition in meat has important consequences in human health [72]. Besides, we observed that among the DE genes there was an overrepresentation of the obesity and the insulin resistance pathways. The results here exposed are particularly interesting because these diseases have a high prevalence and the pig has been described as a suitable biomodel for human lipid-related metabolic diseases [73].

Methods

Animal samples and phenotypes

The IBMAP population was originated by the cross of 3 Iberian boars (Guadyerbas) with 31 Landrace sows [74]. In this study we used 144 animals from the BC1_LD generation obtained by crossing five F1 boars with 26 Landrace sows. Animals were fed *ad libitum* with a cereal-based commercial diet (see [8] for diet details) and slaughtered at 179.8±2.6 days. Animal care and procedures were performed following national and institutional guidelines for the Good Experimental Practices and approved by the Ethical Committee of the Institution (IRTA- Institut de Recerca i Tecnologia Agroalimentàries). Samples of the *Longissimus dorsi* muscle were collected, snap frozen in liquid nitrogen and stored at -80°C until RNA extraction.

A Principal Component Analysis (PCA) was performed for characters related with the FA profile in muscle (see [4] for detailed description of this analysis). Twelve extreme animals were selected according to the first principal component (6 H and 6 L) [4]. Only females were taken into account in order to remove the sex effect on FA composition. The R language [75] was used to perform the statistical analysis of phenotypic mean comparison using a linear model.

RNA isolation

Total RNA was isolated from the *Longissimus dorsi* muscle of 12 samples with RiboPure Isolation of High Quality Total RNA (Ambion, Austin, TX, USA). Total RNA was quantified in a NanoDrop ND-1000 spectrophotometer (NanoDrop products, Wilmington, DE, USA) and Qubit (Invitrogen, Carlsbad, CA, USA). RNA purity and integrity was checked employing a Bioanalyzer-2100 (Agilent Technologies, Inc., Santa Clara, CA, USA). All samples had a RNA Integrity Number (RIN) above 8.5.

Paired-end raw sequences (75 bp) were generated using a Hi-Seq 2000 instrument (Illumina, Inc., San Diego, CA, USA) in CNAG institute (Centro Nacional de Análisis Genómico, Barcelona, España).

Mapping and annotation

We ran FastQC [76] for the quality control. Indexed reads were then mapped to the reference pig genome version 10.2 (Sscrofa10.2) and the annotation database Ensembl Genes 67 [77] using TopHat v2.0.1 [19] with an allowance of two mismatches for each read. The resulting bam files containing the aligned sequences, were subsequently merged with Samtools [78]. Reads were annotated using the intersectBed option of BEDtools [79]. Cufflinks v2.0.2 program [20] was used to assemble the transcripts with a minimum of 10 reads per alignment. Finally, Samtools [78] was employed to compute descriptive statistics.

Gene expression quantification and differential-expression analysis

The number of reads mapping to each gene was determined with the *comp-counts* option in Qualimap v5.0 program [80]. We discarded those genes with a group mean less than 20 counts. We calculated the Pearson correlation coefficient between the mean expression values of the H and L group using the *cor.test* function of R. For the differential expression analysis we used DESeq [25] and EdgeR [24] packages implemented in R. We considered as differentially expressed between H and L groups those genes identified by both programs (DESeq and EdgeR) with a fold change $\geq$1.2 and *P*-value $\leq$0.01, the same parameters used in Corominas *et al.* [5], these case for both programs. FDR was calculated using the R package qvalue [81].

Transposable elements and orthology analysis

We used RepeatMasker version open-3.3.0 [21] with the rm-20120418 database in order to identify repetitive and transposable elements in the pig muscle transcriptome. We used "quick search" and "pig" species options and the Search Engine NCBI/RMBLAST.

Intergenic expressed regions not annotated in the Sscrofa10.2 version assembly were identified using Cuffcompare [82] and extracted using our own Python and R scripts. Novel putative proteins were predicted with Augustus program [22]. Afterwards, using Blast2GO [23], we mapped and annotated the novel protein coding genes. Using BLASTP option (*E*-value hit filter 1.00E-6, annotation cutoff 55, gene ontology (GO) weight 5 and HSP-hit coverage cutoff 0) we checked their orthology with already annotated proteins in *Homo sapiens*, *Bos taurus* and *Sus scrofa* protein databases. The InterProScan specific tool implemented in Blast2GO was employed to refine the functional annotations. With the GO Slim options we selected the relevant GO terms belonging to the cellular component, biological process and molecular function categories. Parameters were set to 10 for the seq filter and 20 for node score filter. Finally the ontology level was set to 3.

Gene ontologies and pathways

The Ingenuity Pathways Analysis software [29] and FatiGO tools from Babelomics 4.3 [28] were used to identify the most relevant biological functions and pathways in which the differentially-expressed genes (between the H and L groups) were involved. IPA, which uses its own private databases, allowed us to identify biological relevant information, identifying overrepresented pathways using the BH multiple testing correction (FDR) at *P*-value $<$0.05, and generating biological networks. For FatiGO, we used KEGG [83] and Reactome [84] databases setting the cut-off FDR $<$0.1. The Mouse Genome Database (MGD) [26] was used in order to identify how mutant alleles driven in mice for the 18 identified genes common in GWAS and RNA-Seq analysis affected the phenotype.

Data Availability

The full data sets have been submitted to NCBI Sequence Read Archive (SRA) under Accession SRP039424, Bioproject: PRJNA240057.

Supporting Information

Figure S1 Network (indirect, score 36) generated by IPA of 35 focus genes corresponding to metabolic disease, lipid metabolism and molecular transport. Node colours indicate gene expression, being the red nodes higher-expressed genes and the green nodes lower-expressed genes in the H group relative to the L group. Colour intensity is related to the degree of expression. Node shapes indicate the biological function of the protein.
(TIF)

Table S1 Percentage of reads mapped for each sample and their localization (exonic, intronic or intergenic) regarding the pig reference genome sequence.
(DOCX)

Table S2 Total number of assembled transcripts with cufflinks.
(DOCX)

Table S3 Description of the repetitive elements identified in the intergenic transcripts of the swine muscle transcriptome.
(DOCX)

Table S4 New predicted novel proteins with Augustus which have orthologous known genes identified with BLASTP option of Blast2GO.
(XLSX)

Table S5 Differentially-expressed genes identified among extreme groups (High and Low) for fatty acid composition in muscle.
(DOCX)

Table S6 Overrepresented categories identified with Babelomics and IPA for the differentially-expressed genes.
(XLSX)

Table S7 Specific functions table identified with IPA for the differentially-expressed genes.
(XLSX)

Table S8 Top networks identified with IPA from the differential expressed genes between High and Low animals.
(XLS)

Acknowledgments

We wish to thank Anna Castelló for RNA isolation and quantification.

Author Contributions

Conceived and designed the experiments: JMF MB. Performed the experiments: APO JMF MB. Analyzed the data: APO YRC MB JE. Contributed reagents/materials/analysis tools: JMF J. Corominas J. Casellas DPM JE YRC. Wrote the paper: APO JMF MB NH.

References

1. Trapnell C, Roberts A, Goff L, Pertea G, Kim D, et al. (2012) Differential gene and transcript expression analysis of RNA-seq experiments with TopHat and Cufflinks. Nat Protoc 7: 562–578. doi:10.1038/nprot.2012.016.
2. Esteve-Codina A, Kofler R, Palmieri N, Bussotti G, Notredame C, et al. (2011) Exploring the gonad transcriptome of two extreme male pigs with RNA-seq. BMC Genomics 12: 552.
3. Chen C, Ai H, Ren J, Li W, Li P, et al. (2011) A global view of porcine transcriptome in three tissues from a full-sib pair with extreme phenotypes in growth and fat deposition by paired-end RNA sequencing. BMC Genomics 12: 448.
4. Ramayo-Caldas Y, Mach N, Esteve-Codina A, Corominas J, Castelló A, et al. (2012) Liver transcriptome profile in pigs with extreme phenotypes of intramuscular fatty acid composition. BMC Genomics 13: 547. doi:10.1186/1471-2164-13-547.
5. Corominas J, Ramayo-Caldas Y, Puig-Oliveras A, Estellé J, Castelló A, et al. (2013) Analysis of porcine adipose tissue transcriptome reveals differences in de novo fatty acid synthesis in pigs with divergent muscle fatty acid composition. Press.
6. Jung WY, Kwon SG, Son M, Cho ES, Lee Y, et al. (2012) RNA-Seq Approach for Genetic Improvement of Meat Quality in Pig and Evolutionary Insight into the Substrate Specificity of Animal Carbonyl Reductases. PLoS ONE 7: e42198. doi:10.1371/journal.pone.0042198.
7. Food and Agriculture Organization (FAO). Available: http://www.fao.org. Accessed 2014 May 20.
8. Serra X, Gil F, Pérez-Enciso M, Oliver M, Vázquez J, et al. (1998) A comparison of carcass, meat quality and histochemical characteristics of Iberian (Guadyerbas line) and Landrace pigs. Livest Prod Sci 56: 215–223. doi:10.1016/S0301-6226(98)00151-1.
9. Wood JD, Enser M, Fisher AV, Nute GR, Sheard PR, et al. (2008) Fat deposition, fatty acid composition and meat quality: A review. Meat Sci 78: 343–358. doi:10.1016/j.meatsci.2007.07.019.
10. Reiter SS, Halsey CHC, Stronach BM, Bartosh JL, Owsley WF, et al. (2007) Lipid metabolism related gene-expression profiling in liver, skeletal muscle and adipose tissue in crossbred Duroc and Pietrain Pigs. Comp Biochem Physiol Part D Genomics Proteomics 2: 200–206. doi:10.1016/j.cbd.2007.04.008.
11. Zhang W, Xiao S, Samaraweera H, Lee EJ, Ahn DU (2010) Improving functional value of meat products. Spec Issue 56th Int Congr Meat Sci Technol 56th ICoMST 15–20 August 2010 Jeju Korea 86: 15–31. doi:10.1016/j.meatsci.2010.04.018.
12. Maharani D, Park H-B, Lee J-B, Yoo C-K, Lim H-T, et al. (2013) Association of the gene encoding stearoyl-CoA desaturase (SCD) with fatty acid composition in an intercross population between Landrace and Korean native pigs. Mol Biol Rep 40: 73–80. doi:10.1007/s11033-012-2014-0.
13. FAO (2008) Fats and fatty acids in human nutrition. Report of expert consultation.
14. Zhang S, Knight TJ, Stalder KJ, Goodwin RN, Lonergan SM, et al. (2009) Effects of breed, sex and halothane genotype on fatty acid composition of triacylglycerols and phospholipids in pork longissimus muscle. J Anim Breed Genet 126: 259–268. doi:10.1111/j.1439-0388.2008.00782.x.
15. Madeira MS, Pires VMR, Alfaia CM, Costa ASH, Luxton R, et al. (2013) Differential effects of reduced protein diets on fatty acid composition and gene expression in muscle and subcutaneous adipose tissue of Alentejana purebred and Large White × Landrace × Pietrain crossbred pigs. Br J Nutr FirstView: 1–14. doi:10.1017/S0007114512004916.
16. Ramayo-Caldas Y, Mercadé A, Castelló A, Yang B, Rodríguez C, et al. (2012) Genome-wide association study for intramuscular fatty acid composition in an Iberian × Landrace cross. J Anim Sci 90: 2883–2893. doi:10.2527/jas.2011-4900.
17. Muñoz M, Rodríguez MC, Alves E, Folch JM, Ibañez-Escriche N, et al. (2013) Genome-wide analysis of porcine backfat and intramuscular fat fatty acid composition using high-density genotyping and expression data.
18. Revilla M, Ramayo-Caldas Y, Castelló A, Corominas J, Puig-Oliveras A, et al. (2014) New insight into the SSC8 genetic determination of fatty acid composition in pigs. Genet Sel Evol In Press.
19. Trapnell C, Pachter L, Salzberg SL (2009) TopHat: discovering splice junctions with RNA-seq. Bioinformatics 25: 1105–1111.
20. Trapnell C, Williams BA, Pertea G, Mortazavi A, Kwan G, et al. (2010) Transcript assembly and quantification by RNA-Seq reveals unannotated transcripts and isoform switching during cell differentiation. Nat Biotechnol 28: 511–515.
21. RepeatMasker version open-3.3.0. Available: http://www.repeatmasker.org. Accessed 2014 May 20.
22. Stanke M, Diekhans M, Baertsch R, Haussler D (2008) Using native and syntenically mapped cDNA alignments to improve de novo gene finding. Bioinformatics 24: 637–644.
23. Conesa A, Götz S, García-Gómez JM, Terol J, Talón M, et al. (2005) Blast2GO: a universal tool for annotation, visualization and analysis in functional genomics research. Bioinformatics 21: 3674–3676. doi:10.1093/bioinformatics/bti610.
24. Robinson M, Oshlack A (2010) A scaling normalization method for differential expression analysis of RNA-seq data. Genome Biol 11: R25.
25. Anders S, Huber W (2010) Differential expression analysis for sequence count data. Genome Biol 11: R106.
26. Mouse Genome Informatics. Available: http://www.informatics.jax.org. Accessed 2014 May 20.
27. Pena RN, Noguera JL, Casellas J, Díaz I, Fernández AI, et al. (2013) Transcriptional analysis of intramuscular fatty acid composition in the longissimus thoracis muscle of Iberian × Landrace back-crossed pigs. Anim Genet: n/a–n/a. doi:10.1111/age.12066.
28. Medina I, Carbonell J, Pulido L, Madeira SC, Goetz S, et al. (2010) Babelomics: an integrative platform for the analysis of transcriptomics, proteomics and genomic data with advanced functional profiling. Nucleic Acids Res 38: W210–W213. doi:10.1093/nar/gkq388.
29. Ingenuity Pathways Analysis software. Available: www.ingenuity.com. Accessed 2014 May 20.

30. Yu K, Shu G, Yuan F, Zhu X, Gao P, et al. (2013) Fatty Acid and Transcriptome Profiling of Longissimus Dorsi Muscles between Pig Breeds Differing in Meat Quality. 9 1: 108–118. doi:doi:10.7150/ijbs.5306.
31. Hamill R, Aslan O, Mullen A, O'Doherty J, McBryan J, et al. (2013) Transcriptome analysis of porcine M. semimembranosus divergent in intramuscular fat as a consequence of dietary protein restriction. BMC Genomics 14: 453.
32. Zhao X, Mo D, Li A, Gong W, Xiao S, et al. (2011) Comparative Analyses by Sequencing of Transcriptomes during Skeletal Muscle Development between Pig Breeds Differing in Muscle Growth Rate and Fatness. PLoS ONE 6: e19774. doi:10.1371/journal.pone.0019774.
33. Putney JW, Bird GSJ (1993) The Inositol Phosphate-Calcium Signaling System in Nonexcitable Cells. Endocr Rev 14: 610–631. doi:10.1210/edrv-14-5-610.
34. Lagace TA, Ridgway ND (2013) The role of phospholipids in the biological activity and structure of the endoplasmic reticulum. Funct Struct Divers Endoplasmic Reticulum 1833: 2499–2510. doi:10.1016/j.bbamcr.2013.05.018.
35. Canovas A, Quintanilla R, Amills M, Pena R (2010) Muscle transcriptomic profiles in pigs with divergent phenotypes for fatness traits. BMC Genomics 11: 372.
36. Quesneville H, Bergman CM, Andrieu O, Autard D, Nouaud D, et al. (2005) Combined Evidence Annotation of Transposable Elements in Genome Sequences. PLoS Comput Biol 1: e22. doi:10.1371/journal.pcbi.0010022.
37. Wilkinson S, Lu ZH, Megens H-J, Archibald AL, Haley C, et al. (2013) Signatures of Diversifying Selection in European Pig Breeds. PLoS Genet 9: e1003453. doi:10.1371/journal.pgen.1003453.
38. Estévez M, Morcuende D, Cava López R (2003) Physico-chemical characteristics of M. Longissimus dorsi from three lines of free-range reared Iberian pigs slaughtered at 90 kg live-weight and commercial pigs: a comparative study. Meat Sci 64: 499–506. doi:10.1016/S0309-1740(02)00228-0.
39. Yendrek C, Ainsworth E, Thimmapuram J (2012) The bench scientist's guide to statistical analysis of RNA-Seq data. BMC Res Notes 5: 506.
40. Soneson C, Delorenzi M (2013) A comparison of methods for differential expression analysis of RNA-seq data. BMC Bioinformatics 14: 91.
41. Antonescu CN, Díaz M, Femia G, Planas JV, Klip A (2008) Clathrin-Dependent and Independent Endocytosis of Glucose Transporter 4 (GLUT4) in Myoblasts: Regulation by Mitochondrial Uncoupling. Traffic 9: 1173–1190. doi:10.1111/j.1600-0854.2008.00755.x.
42. Kubota T, Kubota N, Kadowaki T (2013) The role of endothelial insulin signaling in the regulation of glucose metabolism. Rev Endocr Metab Disord 14: 207–216. doi:10.1007/s11154-013-9242-z.
43. Cao H, Gerhold K, Mayers JR, Wiest MM, Watkins SM, et al. (2008) Identification of a Lipokine, a Lipid Hormone Linking Adipose Tissue to Systemic Metabolism. Cell 134: 933–944. doi:10.1016/j.cell.2008.07.048.
44. Leonardini A, Laviola L, Perrini S, Natalicchio A, Giorgino F (2009) Cross-Talk between PPAR and Insulin Signaling and Modulation of Insulin Sensitivity. PPAR Res 2009. Available: http://dx.doi.org/10.1155/2009/818945.
45. Wilcox G (2005) Insulin and Insulin Resistance. Clin Biochem Rev 2: 19–39.
46. Dai Z, Wu Z, Yang Y, Wang J, Satterfield MC, et al. (2013) Nitric oxide and energy metabolism in mammals. BioFactors 39: 383–391. doi:10.1002/biof.1099.
47. Summers SA, Garza LA, Zhou H, Birnbaum MJ (1998) Regulation of Insulin-Stimulated Glucose Transporter GLUT4 Translocation and Akt Kinase Activity by Ceramide. Mol Cell Biol 18: 5457–5464.
48. Luo J, Sobkiw CL, Hirshman MF, Logsdon MN, Li TQ, et al. (2006) Loss of class IA PI3K signaling in muscle leads to impaired muscle growth, insulin response, and hyperlipidemia. Cell Metab 3: 355–366. doi:10.1016/j.cmet.2006.04.003.
49. Herman MA, Peroni OD, Villoria J, Schon MR, Abumrad NA, et al. (2012) A novel ChREBP isoform in adipose tissue regulates systemic glucose metabolism. Nature 484: 333–338. doi:10.1038/nature10986.
50. Cohen P, Miyazaki M, Socci ND, Hagge-Greenberg A, Liedtke W, et al. (2002) Role for Stearoyl-CoA Desaturase-1 in Leptin-Mediated Weight Loss. Science 297: 240–243. doi:10.1126/science.1071527.
51. Jiang Z, Michal J, Tobey D, Daniels T, Rule D, et al. (2008) Significant associations of stearoyl-CoA desaturase (SCD1) gene with fat deposition and composition in skeletal muscle. Int J Biol Sci 4: 345–351.
52. Renaville B, Prandi A, Fan B, Sepulcri A, Rothschild MF, et al. (2013) Candidate gene marker associations with fatty acid profiles in heavy pigs. Meat Sci 93: 495–500. doi:10.1016/j.meatsci.2012.11.019.
53. Estany J, Ros-Freixedes R, Tor M, Pena RN (2014) A Functional Variant in the Stearoyl-CoA Desaturase Gene Promoter Enhances Fatty Acid Desaturation in Pork. PLoS ONE 9: e86177. doi:10.1371/journal.pone.0086177.
54. Benhamed F, Denechaud P-D, Lemoine M, Robichon C, Moldes M, et al. (2012) The lipogenic transcription factor ChREBP dissociates hepatic steatosis from insulin resistance in mice and humans. J Clin Invest 122: 2176–2194. doi:10.1172/JCI41636.
55. Martinez-Botas J, Anderson JB, Tessier D, Lapillonne A, Chang BH-J, et al. (2000) Absence of perilipin results in leanness and reverses obesity in Leprdb/db mice. Nat Genet 26: 474–479. doi:10.1038/82630.
56. Dalen KT, Dahl T, Holter E, Arntsen B, Londos C, et al. (2007) LSDP5 is a PAT protein specifically expressed in fatty acid oxidizing tissues. Biochim Biophys Acta BBA - Mol Cell Biol Lipids 1771: 210–227. doi:10.1016/j.bbalip.2006.11.011.
57. Bosma M, Sparks LM, Hooiveld GJ, Jorgensen JA, Houten SM, et al. (2013) Overexpression of PLIN5 in skeletal muscle promotes oxidative gene expression and intramyocellular lipid content without compromising insulin sensitivity. Biochim Biophys Acta BBA - Mol Cell Biol Lipids 1831: 844–852. doi:10.1016/j.bbalip.2013.01.007.
58. Furuhashi M, Hotamisligil GS (2008) Fatty acid-binding proteins: role in metabolic diseases and potential as drug targets. Nat Rev Drug Discov 7: 489–503. doi:10.1038/nrd2589.
59. Damon M, Wyszynska-Koko J, Vincent A, Hérault F, Lebret B (2012) Comparison of Muscle Transcriptome between Pigs with Divergent Meat Quality Phenotypes Identifies Genes Related to Muscle Metabolism and Structure. PLoS ONE 7: e33763. doi:10.1371/journal.pone.0033763.
60. Basco D, Blaauw B, Pisani F, Sparaneo A, Nicchia GP, et al. (2013) AQP4-Dependent Water Transport Plays a Functional Role in Exercise-Induced Skeletal Muscle Adaptations. PLoS ONE 8: e58712. doi:10.1371/journal.pone.0058712.
61. Rodríguez A, Catalán V, Gómez-Ambrosi J, García-Navarro S, Rotellar F, et al. (2011) Insulin- and Leptin-Mediated Control of Aquaglyceroporins in Human Adipocytes and Hepatocytes Is Mediated via the PI3K/Akt/mTOR Signaling Cascade. J Clin Endocrinol Metab 96: E586–E597. doi:10.1210/jc.2010-1408.
62. He M, Rutledge S, Kelly D, Palmer C, Murdoch G, et al. (2007) A new genetic disorder in mitochondrial fatty acid beta-oxidation: ACAD9 deficiency. Am J Hum Genet 81: 87–103.
63. Rakhshandehroo M, Knoch B, Müller M, Kersten S (2010) Peroxisome Proliferator-Activated Receptor Alpha Target Genes. PPAR Res 2010. Available: http://dx.doi.org/10.1155/2010/612089.
64. Chegary M, Brinke H te, Ruiter JPN, Wijburg FA, Stoll MSK, et al. (2009) Mitochondrial long chain fatty acid β-oxidation in man and mouse. Biochim Biophys Acta BBA - Mol Cell Biol Lipids 1791: 806–815. doi:10.1016/j.bbalip.2009.05.006.
65. Sung HK, Kim Y-W, Choi SJ, Kim J-Y, Jeune KH, et al. (2009) COMP-angiopoietin-1 enhances skeletal muscle blood flow and insulin sensitivity in mice. Am J Physiol - Endocrinol Metab 297: E402–E409. doi:10.1152/ajpendo.00122.2009.
66. Chen J-X, Stinnett A (2008) Ang-1 Gene Therapy Inhibits Hypoxia-Inducible Factor-1α (HIF-1α)-Prolyl-4-Hydroxylase-2, Stabilizes HIF-1α Expression, and Normalizes Immature Vasculature in db/db Mice. Diabetes 57: 3335–3343. doi:10.2337/db08-0503.
67. Oubaha M, Gratton J-P (2009) Phosphorylation of endothelial nitric oxide synthase by atypical PKCζ contributes to angiopoietin-1–dependent inhibition of VEGF-induced endothelial permeability in vitro. Blood 114: 3343–3351. doi:10.1182/blood-2008-12-196584.
68. Novgorodtseva T, Kantur T, Karaman Y, Antonyuk M, Zhukova N (2011) Modification of fatty acids composition in erythrocytes lipids in arterial hypertension associated with dyslipidemia. Lipids Health Dis 10: 18.
69. Xi Q, Adebiyi A, Zhao G, Chapman KE, Waters CM, et al. (2008) IP3 Constricts Cerebral Arteries via IP3 Receptor–Mediated TRPC3 Channel Activation and Independently of Sarcoplasmic Reticulum Ca2+ Release. Circ Res 102: 1118–1126. doi:10.1161/CIRCRESAHA.108.173948.
70. Bayorh MA, Ganafa AA, Emmett N, Socci RR, Eatman D, et al. (2005) Alterations in Aldosterone and Angiotensin II Levels in Salt-Induced Hypertension. Clin Exp Hypertens 27: 355–367. doi:10.1081/CEH-57423.
71. Merlot E, Vincent A, Thomas F, Meunier-Salaün M-C, Damon M, et al. (2012) Health and immune traits of Basque and Large White pigs housed in a conventional or enriched environment. animal 6: 1290–1299.
72. Webb EC, O'Neill HA (2008) The animal fat paradox and meat quality. 54th Int Congr Meat Sci Technol 54th ICoMST 10-15August 2008 Cape Town South Afr 80: 28–36. doi:10.1016/j.meatsci.2008.05.029.
73. Lunney JK (2007) Advances in Swine Biomedical Model Genomics. Int J Biol Sci 3: 179–184. doi:doi:10.7150/ijbs.3.179.
74. Pérez-Enciso M, Clop A, Noguera JL, Ovilo C, Coll A, et al. (2000) A QTL on pig chromosome 4 affects fatty acid metabolism: evidence from an Iberian by Landrace intercross. J Anim Sci 78: 2525–2531.
75. Ihaka R, Gentleman R (1996) R: A Language for Data Analysis and Graphics. J Comput Graph Stat 5: 299–314. doi:10.2307/1390807.
76. FastQC. Available: http://www.bioinformatics.bbsrc.ac.uk/projects/fastqc/.Accessed 2014 May 20.
77. Ensembl Genes 67. Available: http://www.ensembl.org/info/data/ftp/index.html.Accessed 2014 May 20.
78. Li H, Handsaker B, Wysoker A, Fennell T, Ruan J, et al. (2009) The Sequence Alignment/Map format and SAMtools. Bioinformatics 25: 2078–2079. doi:10.1093/bioinformatics/btp352.
79. Quinlan AR, Hall IM (2010) BEDTools: a flexible suite of utilities for comparing genomic features. Bioinformatics 26: 841–842. doi:10.1093/bioinformatics/btq033.
80. García-Alcalde F, Okonechnikov K, Carbonell J, Cruz LM, Götz S, et al. (2012) Qualimap: evaluating next-generation sequencing alignment data. Bioinformatics 28: 2678–2679. doi:10.1093/bioinformatics/bts503.
81. Storey JD, Tibshirani R (2003) Statistical significance for genomewide studies. Proc Natl Acad Sci 100: 9440–9445. doi:10.1073/pnas.1530509100.
82. Mortazavi A, Williams BA, McCue K, Schaeffer L, Wold B (2008) Mapping and quantifying mammalian transcriptomes by RNA-seq. Nat Methods 5: 621–628.
83. Kyoto Encyclopedia of Genes and Genomes. Available: http://www.genome.jp/kegg/pathway.html. Accessed 2014 May 20.
84. Reactome. Available: http://www.reactome.org. Accessed 2014 May 20.
